# Spore-autonomous fluorescent protein expression identifies meiotic chromosome mis-segregation as the principal cause of hybrid sterility in yeast

**DOI:** 10.1371/journal.pbio.2005066

**Published:** 2018-11-12

**Authors:** David W. Rogers, Ellen McConnell, Jasmine Ono, Duncan Greig

**Affiliations:** 1 Experimental Evolution Research Group, Max Planck Institute for Evolutionary Biology, Plön, Germany; 2 Department of Microbial Population Biology, Max Planck Institute for Evolutionary Biology, Plön, Germany; 3 Department of Genetics, Evolution and Environment, University College London, London, United Kingdom; Duke University Medical Center, United States of America

## Abstract

Genome-wide sequence divergence between populations can cause hybrid sterility through the action of the anti-recombination system, which rejects crossover repair of double strand breaks between nonidentical sequences. Because crossovers are necessary to ensure proper segregation of homologous chromosomes during meiosis, the reduced recombination rate in hybrids can result in high levels of nondisjunction and therefore low gamete viability. Hybrid sterility in interspecific crosses of *Saccharomyces* yeasts is known to be associated with such segregation errors, but estimates of the importance of nondisjunction to postzygotic reproductive isolation have been hampered by difficulties in accurately measuring nondisjunction frequencies. Here, we use spore-autonomous fluorescent protein expression to quantify nondisjunction in both interspecific and intraspecific yeast hybrids. We show that segregation is near random in interspecific hybrids. The observed rates of nondisjunction can explain most of the sterility observed in interspecific hybrids through the failure of gametes to inherit at least one copy of each chromosome. Partially impairing the anti-recombination system by preventing expression of the RecQ helicase *SGS1* during meiosis cuts nondisjunction frequencies in half. We further show that chromosome loss through nondisjunction can explain nearly all of the sterility observed in hybrids formed between two populations of a single species. The rate of meiotic nondisjunction of each homologous pair was negatively correlated with chromosome size in these intraspecific hybrids. Our results demonstrate that sequence divergence is not only associated with the sterility of hybrids formed between distantly related species but may also be a direct cause of reproductive isolation in incipient species.

## Author summary

Different species are kept genetically separated from each other by reproductive isolation, which can result in the production of sterile hybrids. Despite the central role of reproductive isolation in evolutionary biology, the mechanisms underlying hybrid sterility remain controversial. We focus on one potential cause: genome-wide sequence divergence. Sequence divergence can lead to hybrid sterility by interfering with the proper segregation of chromosomes during gamete production, leading to most gametes lacking at least one essential chromosome. While hybrid sterility is known to be associated with mis-segregation in *Saccharomyces* yeasts, quantification of the frequency of these errors has been elusive until now. We use the recently developed technique of spore-autonomous fluorescent protein expression to quantify chromosome segregation errors in gametes produced by both interspecific and intraspecific yeast hybrids. By doing so, we show that segregation is near random in interspecific hybrids, accounting for nearly all of the observed gamete inviability. We also demonstrate that sequence divergence is associated with sterility of hybrids both between distantly related species and between different strains of the same species, providing a general mechanism for reproductive isolation in yeast.

## Introduction

Separate species are often reproductively isolated by intrinsic postzygotic mechanisms. The diverged genomes from two different parental populations may not interact properly when combined in a hybrid, resulting in reduced fertility, reduced viability, or both [1]. Errors during gamete production, particularly problems associated with altered meiotic recombination between diverged genomes, are becoming recognised as a widespread cause of hybrid sterility [2]. While recent work on the contribution of meiotic recombination to reproductive isolation has focused on individual genes [3], seminal studies of the baker's yeast *Saccharomyces cerevisiae* have established genome-wide sequence divergence as a cause of meiotic errors underlying hybrid sterility [4,5]. Sequence divergence between the genomes of different species is thought to decrease the rate of meiotic crossing over through the action of the anti-recombination machinery, which prevents recombination between dissimilar sequences. Low rates of recombination between diverged genomes can cause problems during hybrid meiosis since at least one reciprocal exchange (crossover) event per homologous pair of chromosomes is necessary to ensure correct segregation [4,6]. Failure of homologous chromosomes to segregate correctly (nondisjunction) generates meiotic products (gametic spores) that either contain too many or too few chromosomes, resulting in reduced hybrid spore viability; all *Saccharomyces* chromosomes carry essential genes, and failing to inherit any chromosome results in gamete inviability. The magnitude of the contribution of nondisjunction to postzygotic reproductive isolation in *Saccharomyces* yeasts is not clear because accurate measurements of nondisjunction rates are not currently available. Attempts to quantify the rate of nondisjunction in *Saccharomyces* hybrids have relied on the analysis of colonies grown from single spores (obtained by tetrad dissection or random spore analysis). These studies have suffered from numerous methodological biases stemming from three principal sources: (1) chromosome loss during mitotic growth, (2) limited sensitivity of the techniques used for measuring ploidy, and (3) the inability to genotype inviable spores. As a result, neither extra copies of chromosomes nor missing copies can be accurately quantified using single-spore–derived colonies.

Extra copies of chromosomes (disomes) inherited by haploid spores during meiosis are highly unstable and prone to mitotic loss following germination [7]. Disomes generally do not have large effects on the probability of spore germination in *S*. *cerevisiae* [8,9] but do tend to reduce mitotic growth rates relative to eusomic strains [10,11]. As a result, in a growing colony derived from a single disomic spore, mutants that lose one copy of a disomic chromosome will gain a growth advantage over disomic cells and increase in frequency, potentially becoming the dominant type in a heterogeneous colony. Moreover, by generating stoichiometric imbalances in proteins important for mitotic segregation, aneuploidy itself might increase genomic instability [12]. Disome loss has been demonstrated using the meiotic products of triploid yeast [8]. In a triploid meiosis, each chromosome will be inherited as a disome by two of the spores in the resulting tetrad and as a monosome (single copy) by the other two spores (resulting in an average of 8 disomes per spore). However, when chromosomes are counted in colonies derived from the four spores of a four-viable–spore tetrad, disomes are underrepresented relative to monosomes. The magnitude of the deficit depends on the sensitivity of the assay used to detect the presence or absence of each chromosome [8,13,14]. When chromosome loss occurs early in the growth of a colony or is associated with a large increase in growth rate, the lost chromosome may be present in only a small proportion of cells in the colony when analysed, and correct diagnosis of disomy in the founding spore would require a highly sensitive assay. For example, St. Charles and colleagues [8] detected only 853 of 960 expected chromosomes in 10 four-viable–spore triploid-derived tetrads using a comparative genome hybridisation assay, corresponding to an average of 5.3 disomes per spore. When a more sensitive PCR-based assay was used, they detected 945 of 960 expected chromosomes, corresponding to an average of 7.6 disomes per cell. Chromosome loss during mitotic growth means that measuring disome frequency in single-spore–derived colonies will always underestimate the true magnitude of the problem.

The frequencies of disomes in the gametes of yeast hybrids generated by crossing *S*. *cerevisiae* and *S*. *paradoxus* have been estimated using multiple techniques with inconsistent results. Hunter and colleagues [4] reported highly variable frequencies of disomes for different chromosomes, ranging from 0% (chromosome [Chr] VI) to 27.2% (Chr II) with a mean of 12.2%, corresponding to 1.95 disomes per cell (mean number of disomes = 16 × mean frequency of nondisjunction). Disome frequency was measured by karyotyping randomly selected single-spore-derived colonies using a pulsed field gel electrophoresis (PFGE) technique that allows visual identification of disomes as either two separate bands or a single band twice as bright as that corresponding to a monosome. When disomes are present in only a small proportion of cells in a colony (because of mitotic loss), the sensitivity of detection will depend on whether the interspecific homologs migrate together or as two distinct bands, with the latter case affording much greater sensitivity. Indeed, Hunter and colleagues [4] reported high disome frequencies for the three homologs that migrate as separate bands (mean for Chr I, II, and VIII = 22.3% or 3.57 disomes per spore) compared to six chromosomes that comigrate (mean for Chr III, VI, IX, X, XI, and XIV = 7.1% or 1.14 disomes per spore). A more sensitive PCR-based approach was used to estimate frequencies of extra copies of all 16 chromosomes in spores derived from the same *S*. *cerevisiae* × *S*. *paradoxus* cross by Greig and colleagues [15], who found that *S*. *cerevisiae* chromosomes were over-represented in colonies derived from random spores by 31% (compared to the random-segregation expectation of 33% of viable spores, see below). They inferred that this degree of over-representation was evidence of high numbers of disomes (an average of 4.96 disomes per spore) caused by near-random segregation of homologous chromosomes in hybrid meiosis. Unfortunately, the lack of *S*. *paradoxus* genomic sequences at the time of these experiments made it impossible to confirm that the over-representation of *S*. *cerevisiae* sequences was actually attributable to the presence of disomes. Instead, Greig and colleagues [15] analysed their hybrid-spore–derived colonies by PFGE—looking only at chromosomes (I, II, and VIII) that could be resolved as separate bands—and found similar disome frequencies to the average value of 22.3% reported by Hunter and colleagues [4] with an average of 25.7% (or 4.10 disomes per spore). Thus, PFGE results are consistent with a relatively low overall disome frequency when disomes are scored based on both the number and intensity of bands [4] but also with near-random segregation when only disomes migrating as two distinct bands are counted [15]. More recently, Kao and colleagues [16] used a comparative genome hybridization assay on random-viable-spore–derived colonies and detected an average of 2.29 disomes per spore (14.3%), but the sensitivity of their assay is difficult to assess.

Nondisjunction frequencies are further biased by the inability to score dead spores. If a chromosome pair segregates randomly, half the time it will segregate correctly and half the time incorrectly. Therefore, under random segregation of a chromosome, 50% of spores should exhibit the consequences of nondisjunction: 25% will contain two copies of the chromosome (disomes) and 25% will contain no copies of the chromosome (nullosomes) [6]. However, since the 25% of spores that fail to inherit at least one copy cannot be scored, one third of observed spores should contain disomes and two thirds should contain monosomes, resulting in a maximum observed nondisjunction frequency of only 33%. Additionally, any disome (such as Chr VI [10]) or combination of disomes that reduces the likelihood of germination will be underrepresented in analyses of viable-spore–derived colonies. However, apart from Chr VI, there is little evidence that the presence of disomes or combinations of disomes has large effects on the likelihood of spore germination [8,9].

Mitotic disome loss, insensitive disome detection, and the inability to score dead spores mean that karyotype data from the colonies produced by viable spores can only be used to determine a lower bound for nondisjunction rates, preventing the causes of hybrid sterility from being quantified. Accurate assessment of nondisjunction rates is therefore crucial to understanding the contribution of meiotic mis-segregation to postzygotic reproductive isolation in yeast. Under the simple assumption that spores inheriting zero copies of any chromosome are inviable, but disomes do not affect the likelihood of germination, random segregation of homologous chromosomes is sufficient to explain all of the observed inviability of *S*. *cerevisiae* × *S*. *paradoxus* hybrid spores: the probability of inheriting at least one copy of each chromosome would be 0.75^16^ or 1.0%, equivalent to the measured viability of spores produced by these interspecific hybrids [4,17]. Consequently, the high rate of mis-segregation reported by Greig and colleagues [15] would explain nearly all of the infertility observed in interspecific hybrids (0.763^16^ = 1.3% viability). The lower rate reported by Hunter and colleagues [4] would allow much higher spore viability (0.891^16^ = 15.9% viability) than observed, meaning that other mechanisms such as toxic disomy or additional forms of hybrid incompatibility must be invoked to explain the low fertility of interspecific hybrids. Accurate assessment requires a sensitive assay that measures the frequency of disomes in spores directly (preferably in tetrads themselves) and independently of spore viability. Here, we have used a spore-autonomous fluorescent protein expression assay developed by Thacker and colleagues [18] based on a similar system developed in *Arabidopsis* [19] to accurately measure meiotic mis-segregation in yeast hybrids.

## Materials and methods

### Strain construction

The spore-autonomous expression system developed by Thacker and colleagues [18] (received as a kind gift from Scott Keeney) expresses a fluorescent protein (GFP, mCerulean, or tdTomato) under the control of the spore-autonomous *YKL050c* promoter and the *PGK1* terminator. Each fluorescent protein is paired with a promoter/terminator combination from a different *Saccharomyces* species (GFP: *S*. *mikatae*; mCerulean: *S*. *bayanus*; tdTomato: *S*. *kudriavzevii*). Unfortunately, we found that although these constructs resulted in spore-autonomous expression when integrated into the genome of the *S*. *cerevisiae* strain used here (Y55), when integrated into the genome of our *S*. *paradoxus* strain (N17), they resulted in very weak expression in both spores and vegetative diploids. Swapping the heterologous *YKL050c* promoter for the species-specific version resulted in some improvement (see S1 Text and S1 Fig), but expression in *S*. *paradoxus* and hybrid spores remained weak. We therefore swapped the *YKL050c* promoter for a different spore-autonomous promoter: the *DIT1* (*YDR403w*) promoter [20]. The *DIT1* gene is expressed exclusively in sporulating cells, with transcripts first appearing 8–10 hours after transfer to sporulation medium and reaching maximal levels after 12–14 hours, corresponding to the time of prospore enclosure. Expression of both GFP and tdTomato under a strain-specific *DIT1* promoter resulted in strong spore-specific and spore-autonomous fluorescence in *S*. *cerevisiae*, *S*. *paradoxus*, and F1 hybrids.

Fluorescent protein expression was placed under control of the *DIT1* promoter by replacing the endogenous *DIT1* ORF in the desired *S*. *cerevisiae* or *S*. *paradoxus* haploid (S4 Fig) with the entire construct developed by Thacker and colleagues [18] except for the *YKL050c* promoter (i.e., from the start codon of GFP or tdTomato to the end of the *URA3* or *LEU2* cassette, respectively). These constructs, P_DIT1__GFP_URA3 and P_DIT1__RFP_LEU2, were then integrated into selected loci on each chromosome to allow analysis of segregation of all chromosome pairs (S4 Fig). Full details of strain construction are provided in the S1 Text.

### Scoring meiotic segregation in parents and hybrids

**Fig 1:**
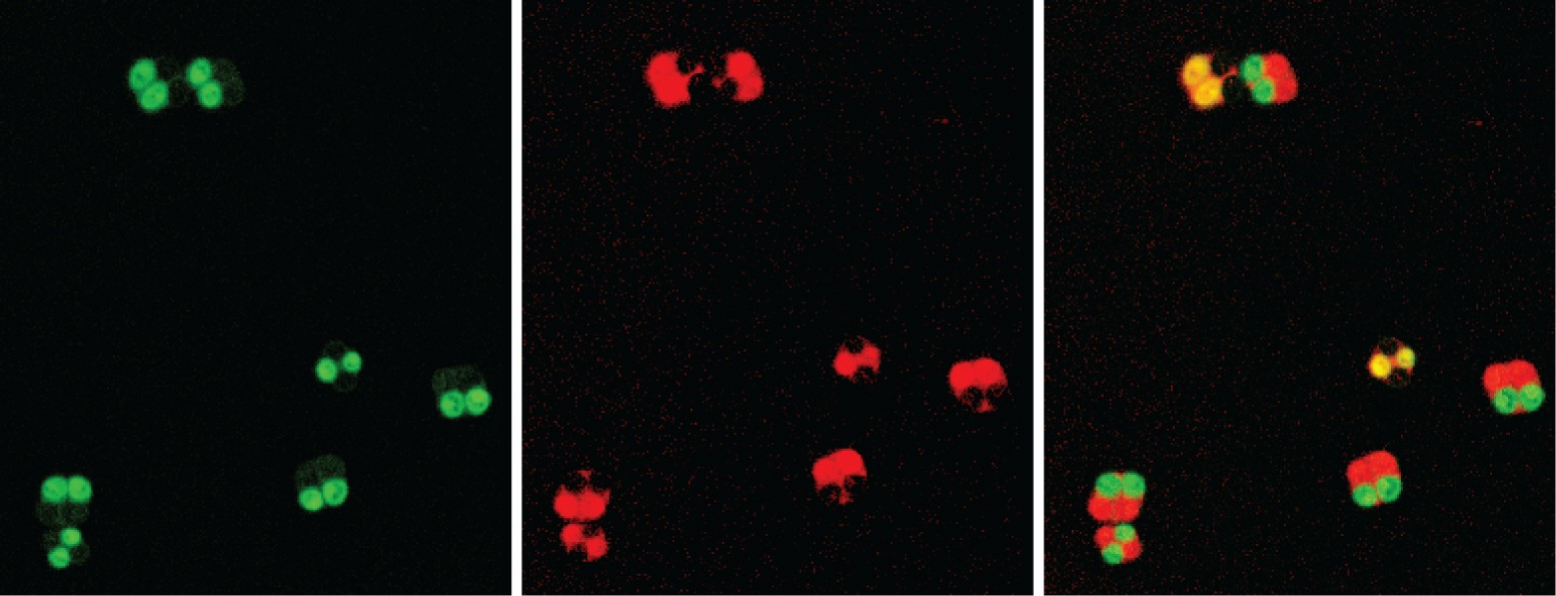
Spore-autonomous expression of fluorescent proteins under the *DIT1* promoter. Hybrid diploids were created by crossing a MATα strain with a single chromosome marked with *pDIT1*_*tdTomato* with a MATa strain marked with *pDIT1*_*GFP* at the same position on the same chromosome. Sporulation of these diploids generates asci containing 4 spores (tetrads). Correct segregation of homologous chromosomes during meiosis I results in tetrads containing 2 GFP-expressing spores and 2 tdTomato-expressing spores. Under meiosis I nondisjunction, homologous chromosomes fail to segregate, resulting in 2 spores expressing both GFP and tdTomato and 2 spores expressing neither. Left panel = GFP fluorescence; middle panel = tdTomato fluorescence; right panel = overlay. Here, we show 7 tetrads produced by sporulating YDP1480 × YDP1559 hybrid diploids, 5 of which show correct segregation and 2 of which show nondisjunction (yellow spores in overlay). GFP, green fluorescent protein; tdTomato, tandem dimer Tomato fluorescent protein.

Sporulation was performed as described by Thacker and colleagues [18]. Briefly, prototrophic diploid *S*. *cerevisiae*, *S*. *paradoxus*, or (inter- or intraspecific) F1 hybrids were inoculated into 5 mL of liquid 1% YPA presporulation medium (1% Difco yeast extract, 2% Bacto peptone, 1% potassium acetate) and grown overnight at 30°C. Cells in 1 mL of each overnight culture were harvested by centrifugation, washed in water, resuspended in 2 mL liquid sporulation medium (2% potassium acetate), and incubated with shaking for 24 h. Slides were prepared by mounting 10 μL of sporulated cultures under a coverslip, sealing with nail polish, and squashing gently to flatten tetrads. We only scored tetrads for which all four spores were present, clearly visible, and easily distinguishable from those of neighbouring tetrads. Fluorescence was manually scored in tetrads using either a Zeiss Axio Scope A.1 (Zeiss, Jena, Germany) equipped with Filter Sets 38HE (GFP) and 43HE (tdTomato) and a Zeiss EC Plan-NEOFLUAR 100× oil immersion objective or a Zeiss Axio Imager M1 equipped with Filter Sets 38 (GFP) and 20 (tdTomato) and a Zeiss EC Plan-NEOFLUAR 40×/0.75 Ph2 objective. Each tetrad was identified under visible light, scored for GFP and then tdTomato (or vice versa), and then double-checked. The vast majority of tetrads either had 2 green spores and 2 red spores (correct segregation) or 2 nonfluorescent spores and 2 spores that were both green and red (meiosis I nondisjunction, Fig 1). A total of 4.0% (2,062/51,112) of tetrads exhibited other fluorescence patterns. Of these, the most frequent patterns were 3 spores of one colour and 1 of the other (likely a gene conversion event between the fluorescent markers) or 2 spores of one colour, 1 of the other, and 1 nonfluorescent spore (likely a meiosis II nondisjunction event). Because these patterns were not consistent with meiosis I nondisjunction, they were included in the total tetrad counts as having segregated correctly. Our estimates of the total nondisjunction frequency may therefore be slightly conservative.

## Results and discussion

**Fig 2:**
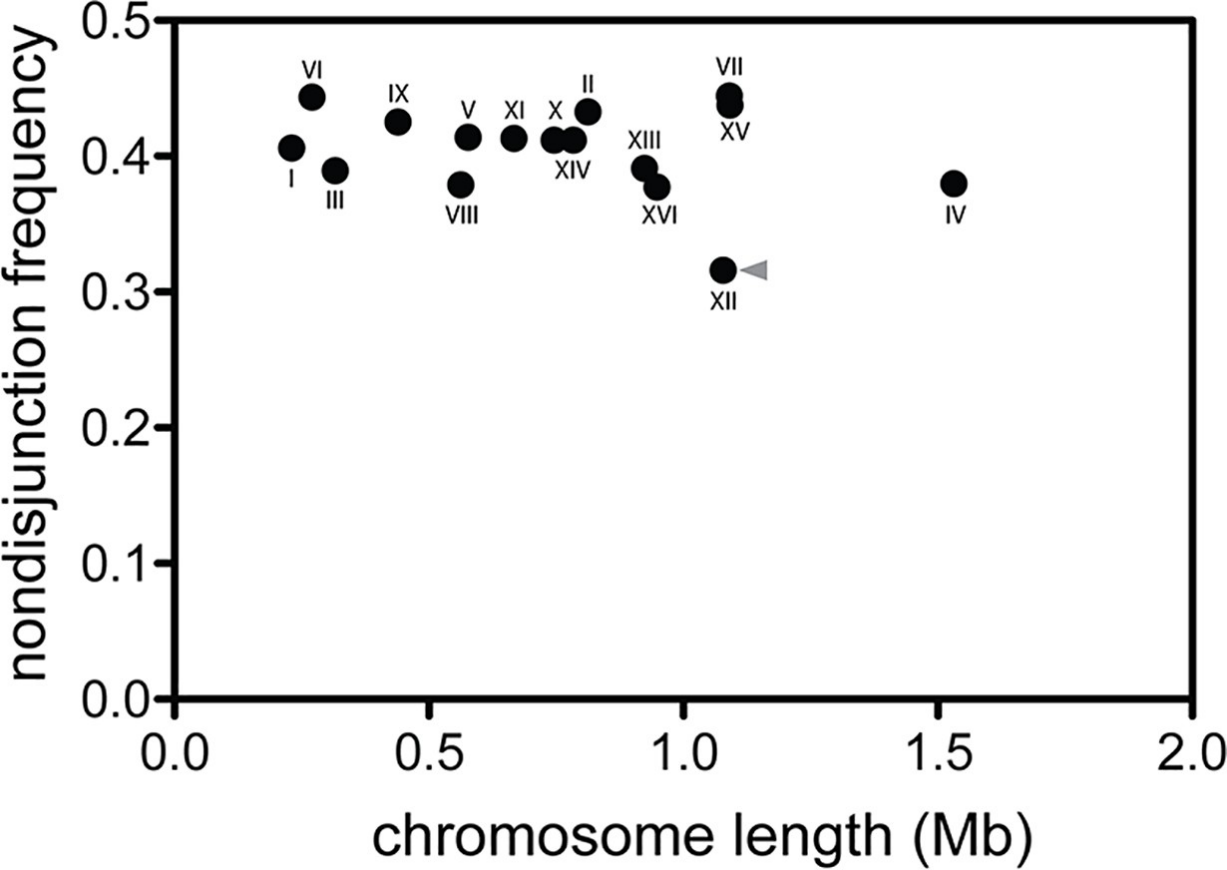
An interspecific cross between *S*. *cerevisiae* and *S*. *paradoxus* exhibits extremely high rates of meiosis I nondisjunction. Black circles represent the nondisjunction rate for each of 16 homologous chromosome pairs. Underlying data can be found in S1 Data. Random segregation would correspond to a nondisjunction frequency of 0.5, while perfect segregation would correspond to a nondisjunction frequency of 0.0. Chromosome lengths represent the total amount of alignable sequence per chromosome pair between the genomes of the two species. Length estimates were obtained using the complete genomes of *S*. *cerevisiae* strain SK1 (instead of Y55) and *S*. *paradoxus* strain CBS432 (instead of N17) because these are the most closely related strains for which end-to-end assemblies are available [22]. The alignable regions of these two genomes are 87.8% identical (9,784,711 out of 11,146,833 sites). The length of Chr XII (indicated by the grey arrowhead) omits the tandem rDNA repeats, which could not be assembled; the true size of Chr XII may be twice as long as presented. Chr, chromosome; rDNA, ribosomal DNA.

We observed extremely high levels of chromosomal nondisjunction during meiosis in interspecific hybrids formed between *S*. *cerevisiae* strain Y55 and *S*. *paradoxus* strain N17 (Fig 2). On average, each chromosome pair failed to segregate during meiosis I in 40.3% of hybrid sporulation events, with a total of 6,276 out of 15,588 tetrads exhibiting nondisjunction. If spores containing at least one copy of each chromosome are viable, the observed nondisjunction rates would result in a spore viability of 2.7%, comparable to approximately 1% observed experimentally [4,17]. Thus, the loss of chromosomes due to meiosis I nondisjunction can explain nearly all of the infertility in hybrids between these two species. Nondisjunction was extremely rare in the nonhybrid parental strains, occurring in only 0.15% (7/4,527) of tetrads in the *S*. *cerevisiae* Y55 parent and 0.06% (3/5,145) of tetrads in the *S*. *paradoxus* N17 parent. The observed parental nondisjunction frequencies are similar to previously reported values [18,21], indicating our genetic manipulations have not inflated meiotic mis-segregation. We observed similar levels of nondisjunction to those reported above using species-specific *YKL050c* promoters across five tested chromosomes (43.8% in N17 × Y55 hybrids compared to 41.0% for the same five chromosomes using the *DIT1* promoter), although this system was more difficult to score (S1 Fig).

**Fig 3:**
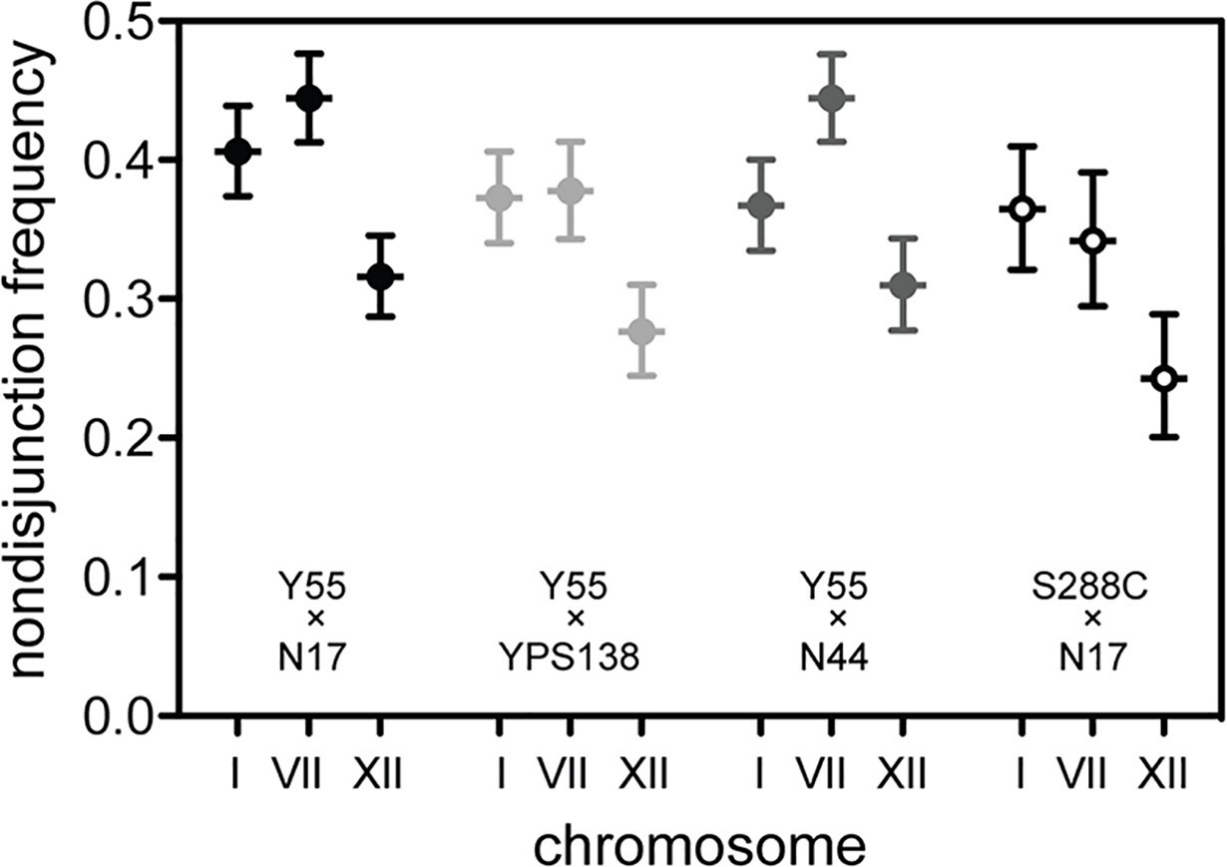
Chromosome XII shows reduced nondisjunction in interspecific hybrids. Chr XII exhibited a markedly lower nondisjunction rate than did other chromosomes in interspecific hybrids between *S*. *cerevisiae* strain Y55 and *S*. *paradoxus* strain N17 (data reproduced from Fig 2). We confirmed this result by examining the segregation of three chromosome pairs (I, VII, and XII) in crosses between *S*. *cerevisiae* strain Y55 and two other *S*. *paradoxus* strains from different clades: the American B strain YPS138 and the Far Eastern strain N44, as well as a cross between *S*. *paradoxus* strain N17 and a second *S*. *cerevisiae* strain, the laboratory model S288C. Underlying data can be found in S1 Data. Error bars represent binomial 95% confidence intervals calculated using JavaStat (http://statpages.info/confint.html). Chr, chromosome.

Most chromosomes exhibited similar levels of mis-segregation, and no relationship was observed between the rate of nondisjunction and chromosome length (Fig 2: Spearman rank correlation r_s_ = −0.085, *P* = 0.754). One marked exception was Chr XII, which segregated much more reliably than did any other chromosome (nondisjunction rate of only 31.6%). To confirm the generality of this lower rate of mis-segregation of Chr XII in interspecific hybrids, we examined the segregation of Chr I, VII, and XII in crosses between *S*. *cerevisiae* strain Y55 and the additional *S*. *paradoxus* strains YPS138 and N44 and in a cross between *S*. *paradoxus* strain N17 and the additional *S*. *cerevisiae* strain S288C (Fig 3). We found that Chr XII had lower rates of nondisjunction than did Chr I and VII in all interspecific hybrids tested. Chr XII contains a single long ribosomal DNA (rDNA) tract in *Saccharomyces* yeasts consisting of an uninterrupted stretch of 150–200 identical or nearly identical approximately 9,100 base pair head-to-tail tandem repeats [23]. Recombination is very frequent in the rDNA of *S*. *cerevisiae* [24], and this region has been shown to promote mitotic chromosome segregation fidelity [25]. It is intriguing to speculate that the large size and repetitive nature of the rDNA may promote crossovers between hybrid sister chromosomes, ensuring correct segregation during meiosis. The 18S, 5S, and 25S ribosomal-RNA–encoding regions, but not the intergenic spacers, are almost perfectly conserved between *S*. *cerevisiae* and *S*. *paradoxus* [26–28], providing an excellent template for double-strand–break repair. If anti-recombination is responsible for the high rates of nondisjunction in yeast hybrids, it seems reasonable that the long tracts of identity associated with rDNA would help to rescue segregation on Chr XII as shown in Fig 3.

The failure of homologous chromosomes to segregate correctly during meiosis I in hybrids is often attributed to the activity of mismatch repair and anti-recombination systems that prevent recombination between divergent DNA sequences [4,5,21]. We used our spore-autonomous fluorescent markers to compare the recombination rate between two loci on Chr XI in *S*. *cerevisiae* strain Y55, *S*. *paradoxus* strain N17, and their interspecific hybrid as described by Thacker and colleagues [18]. We calculated the map distance between YKR005c and YKL050c, located approximately 100 kb apart on Chr XI, to be 36.1 cM in Y55 and 32.9 cM in N17 but only 0.4 cM in their hybrid (S3 Fig). Thus, at least in this region of the genome, the recombination rate in the interspecific hybrids is only 1% of that in the parents. Given meiosis in *S*. *cerevisiae* typically involves 90 crossovers [29], extrapolating our observation across the genome suggests that interspecific hybrids experience only about 1 crossover per meiosis—a value comparable to that of 2.7 crossovers per viable spore found in hybrids of the same two species [16]. Intraspecific hybrids between closely related parents have previously been shown to suffer from reduced recombination as well. In a genome-wide screen, Martini and colleagues [30] found that hybrids between *S*. *cerevisiae* strains S288C and SK1 (a very close relative of Y55 and about 0.7% diverged from S288C) underwent an average of only 73 crossovers per meiosis, but disrupting the anti-recombination gene *MSH2* restored the number of crossovers to the level seen in the parents (>90).

**Fig 4:**
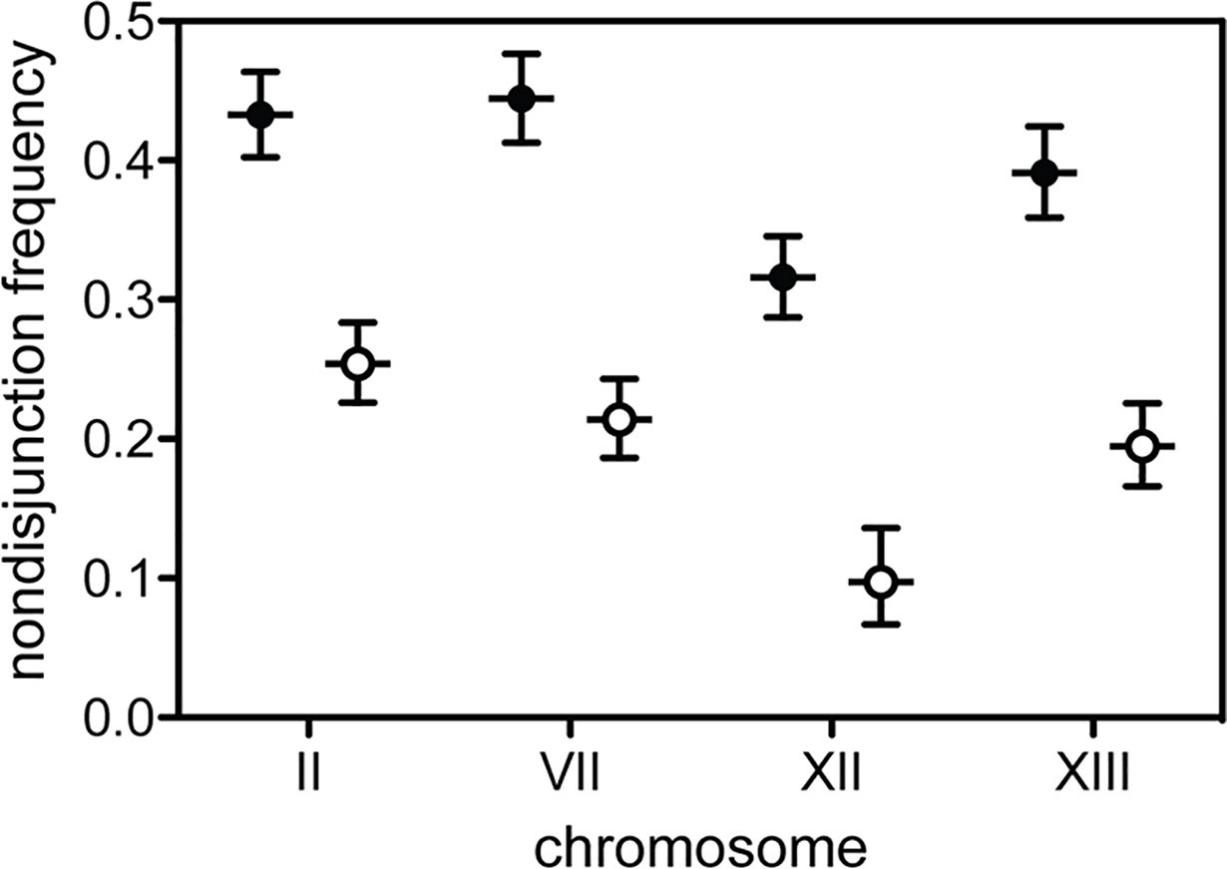
Impaired anti-recombination reduces meiosis I nondisjunction in interspecific hybrids. Eliminating expression of the RecQ helicase Sgs1 during meiosis (open symbols) lowered the rate of nondisjunction to approximately half of that seen in wild-type interspecific N17 × Y55 hybrids (closed symbols) for three of the tested chromosomes (II, VII, and XIII) and approximately one third for Chr XII. Underlying data can be found in S1 Data. Error bars represent binomial 95% confidence intervals calculated using JavaStat (http://statpages.info/confint.html). Chr, chromosome.

To further investigate the consequences of impaired anti-recombination on meiotic nondisjunction in our interspecific hybrids, we used strains in which expression of the RecQ helicase *SGS1* was placed under the control of the *CLB2* promoter, which is strongly repressed during meiosis [31]. Sgs1 is required for rejection of homeologous recombination [32], and the *pCLB2*_*SGS1* construct has previously been found to reduce nondisjunction in partial hybrids between *S*. *cerevisiae* and *S*. *paradoxus* [33]. Consistent with these results, we found substantially reduced nondisjunction of four tested chromosomes (II, VII, XII, and XIII) in interspecific hybrids with impaired anti-recombination relative to wild-type controls (Fig 4). Chr XII exhibited the largest response to impairing anti-recombination in hybrids, with a 3.2-fold improvement in segregation compared to approximately 2-fold for the others. Parental strains exhibited extremely low levels of nondisjunction whether anti-recombination was impaired or intact. Our results add to the mounting evidence that anti-recombination is largely responsible for the sterility of these interspecific hybrids. Indeed, recent work has demonstrated that disabling the activity of multiple components of the anti-recombination machinery during meiosis results in a large improvement in *S*. *cerevisiae* × *S*. *paradoxus* hybrid spore viability (from <1% to >30%) associated with a decrease in disome frequency and an increase in crossover frequency of similar magnitudes [34].

**Fig 5:**
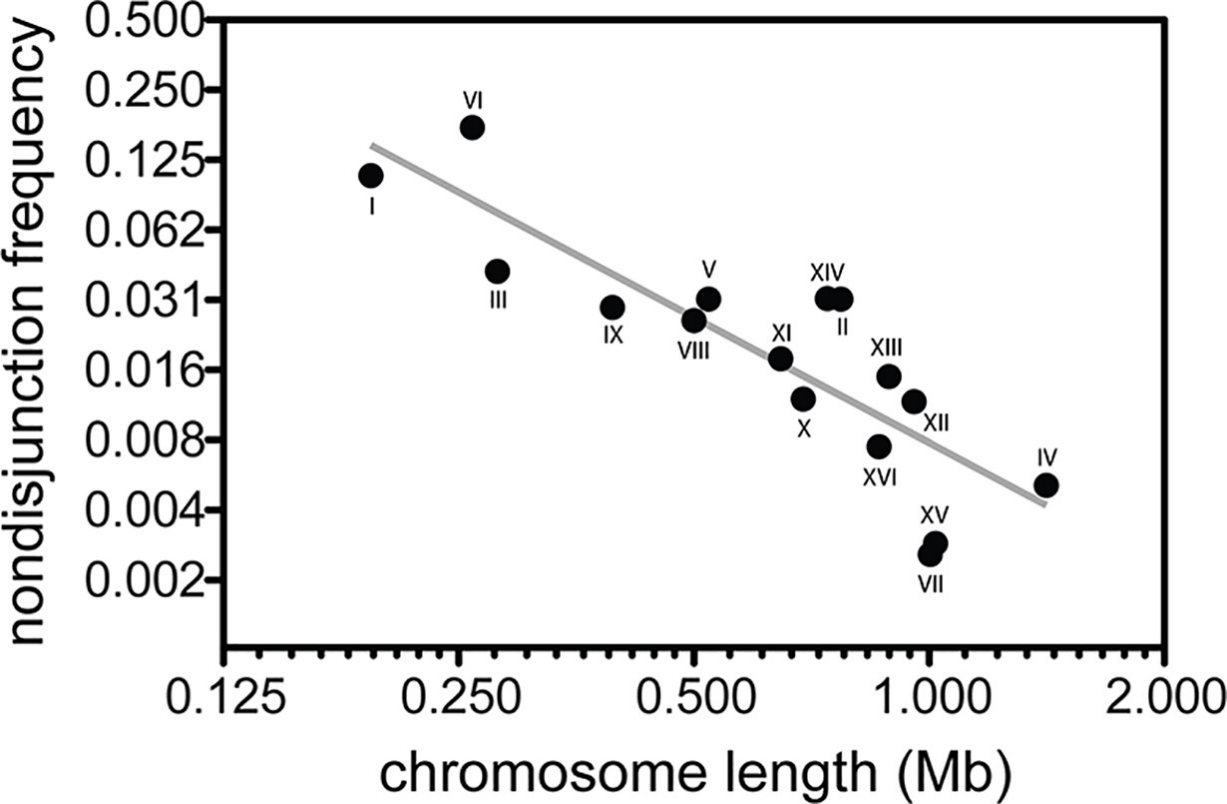
Short chromosomes fail to disjoin at higher rates than long chromosomes in an intraspecific *S*. *paradoxus* hybrid. Circles represent the nondisjunction frequency for each of the 16 homologous chromosome pairs in hybrids formed between the *S*. *paradoxus* strains N17 and N44. Underlying data can be found in S1 Data. Chromosome lengths represent the total amount of alignable sequence per chromosome pair between the genomes of each species. Length estimates were obtained using the complete genomes of *S*. *paradoxus* strains CBS432 (instead of N17) and N44 [22]. The alignable regions of these two genomes are 98.6% identical (11,358,261 identical sites out of 11,516,349 total sites). The grey line represents the line of best fit: log nondisjunction = −2.12 − 1.79(log length).

The relevance of meiotic nondisjunction of homologous chromosomes in *S*. *cerevisiae* × *S*. *paradoxus* hybrids to models of speciation has been questioned because of the high genetic divergence between these species (12.2% sequence divergence); many reproductively isolated species are much less diverged [1,35]. Despite these objections, deletion of the mismatch repair gene *MSH2* has been found to improve the fertility—relative to their parents—of intraspecific hybrids formed by crossing different strains of *S*. *paradoxus* [21]. Indeed, very small numbers of mismatches can greatly reduce the rate of recombination in *Saccharomyces*: a single mismatch in a 350-base pair sequence was found to reduce homologous recombination 3- to 4-fold, and two to three additional mismatches reduced the frequency of recombination 9-fold relative to identical sequences [36]. Mismatch-repair–controlled anti-recombination is thought to cause the crossover frequency to fall exponentially with sequence divergence, and the rate of decline is strongest for near-identical sequences [36]. Consequently, meiosis I nondisjunction should be observable in hybrids formed between closely related populations. To determine the importance of meiotic nondisjunction to the fertility of intraspecific hybrids, we examined segregation in the spores of a diploid formed by crossing *S*. *paradoxus* strains from the European and Far Eastern clades [37]: N17 and N44 (1.4% sequence divergence). We found this cross produced 63.9% viable spores (253 viable out of 396 total spores), slightly lower than the value of 77.1% reported in [38] for a similar cross (CBS432 × N44). The average nondisjunction rate in intraspecific hybrids was much lower than observed in interspecific hybrids, with only 3.4% of homologous pairs failing to segregate (Fig 5). If, as above, we assume that all spores inheriting at least one copy of each chromosome are viable, then the observed levels of nondisjunction would result in 75.7% spore viability. Therefore, as observed in interspecific hybrids, nondisjunction can explain most of the infertility in hybrids formed between much more closely related strains (S2 Fig). We confirmed the occurrence of nondisjunction in intraspecific hybrids by examining segregation of Chr I in a second *S*. *paradoxus* cross (N17 × YPS138, 3.5% genetic divergence), which showed a similar nondisjunction frequency (10.8%) to that seen in the N17 × N44 hybrids (10.7%, S1 Data, S2D Fig). Even a cross between two very closely related *S*. *cerevisiae* strains (Y55 × S288C, 0.6% genetic divergence) exhibited detectable levels of Chr I nondisjunction (1.1%; none was observed for the Y55 parent).

In contrast to the roughly uniform nondisjunction rates for different chromosomes in the interspecific hybrid, we observed a strong negative correlation between chromosome length and the rate of nondisjunction in the intraspecific hybrid (Spearman rank correlation r_s_ = −0.860, *P* < 0.0001); the relationship is log–log linear with a slope of −1.79 (Fig 5). This negative relationship was expected since crossover frequency is tightly associated with chromosome size in *S*. *cerevisiae* intraspecific hybrids (S288C × YJM789, SNP-based genomic distance 99.54% identical [37]) in which the number of crossovers per chromosome increases by approximately 0.6 per 100 kb of chromosome length [29,39]. If longer chromosomes are more likely to experience at least one crossover event, then they should exhibit lower rates of nondisjunction. Indeed, we found that only Chr I and VI exceeded a nondisjunction frequency of 5% in the intraspecific hybrid; these two chromosomes also have the highest probabilities of exhibiting zero crossovers in homozygous parents [29]. We cannot explain why the negative relationship between nondisjunction and chromosome length is so clear in our analysis of an intraspecific hybrid (Fig 5) but undetectable in our analysis of an interspecific hybrid (Fig 2). Given the very low frequency of recombination in interspecific hybrids, it is possible that our analysis simply lacked the power to detect a weak negative correlation. Alternatively, it is also possible that rare crossovers in these hybrids are more likely to occur on longer chromosomes but that this is balanced by a bias in crossover-independent mechanisms towards rescuing segregation of shorter nonexchange chromosomes [29].

The levels of nondisjunction we observed in intraspecific and interspecific meiosis are likely attributable to sequence divergence between the two parents. A strong negative relationship between *S*. *paradoxus* hybrid spore viabilities and parental genetic distances was reported by Liti and colleagues [38]. After correction for inviability associated with chromosomal rearrangements, they found this relationship was best fit by an exponential decay function. In contrast, examining hybrids formed between strains of *S*. *cerevisiae*—a less genetically diverse group than *S*. *paradoxus*—Hou and colleagues [40] reported no correlation between hybrid spore viabilities and parental genetic distances. However, if the spore viabilities measured by Hou and colleagues [40] are adjusted to account for the effects of chromosomal rearrangements as done by Liti and colleagues [38], a strong negative correlation immediately becomes apparent (S2 Fig). Indeed, the relationship is similar to that reported for *S*. *paradoxus* hybrids (and consistent with the results presented here for nondisjunction in the N17 × N44 hybrid): spore viability decreases by roughly 1.25% for each 0.1% parental sequence divergence. We further show a similar negative relationship in a separate *S*. *cerevisiae* data set collected for the 100-genomes strains (S2 Fig, [41]). It therefore seems entirely plausible that small amounts of sequence divergence can contribute to reproductive isolation and incipient speciation.

We have shown that meiosis I nondisjunction alone can explain nearly all of the sterility of the yeast hybrids studied here, simply because of spores failing to inherit essential chromosomes. If the unexplained spore inviability can be attributed to disomy arising from nondisjunction, then no other mechanism would be necessary to explain yeast hybrid sterility. The magnitude of the effect of meiotic mis-segregation on hybrid sterility has previously been hard to gauge because of technical difficulties in accurately quantifying nondisjunction rates. Moreover, since previous evidence for the association between nondisjunction and postzygotic reproductive isolation was restricted to crosses between highly diverged species (*S*. *cerevisiae* and *S*. *paradoxus*), a major role of sequence divergence as a cause of hybrid sterility between closely related populations has often been dismissed [1,35,42,43]. Here, we show that nondisjunction can explain most of the hybrid fertility defect observed not only for interspecific crosses but also for an intraspecific cross between much more closely related strains. These results suggest that even small amounts of sequence divergence can directly contribute to postzygotic reproductive isolation, consistent with the negative correlation between hybrid spore viability and parental sequence divergence observed both within and between species (S2 Fig, [38,44,45]).

We do not mean to suggest that anti-recombination is the sole mechanism underlying postzygotic reproductive isolation in yeast. Chromosomal rearrangements can clearly contribute to the genetic isolation of *Saccharomyces* populations [40,46], and we have intentionally chosen collinear strains to remove these effects. Furthermore, although there is no evidence for lethal genic incompatibilities between the genomes of *S*. *cerevisiae* and *S*. *paradoxus* [16,47], negative epistasis does reduce hybrid viability between these two species in particular environments [3,48]. It is also possible that either the number of spores per tetrad [49] or the efficiency with which diploids sporulate might be affected by negative epistasis between hybrid genomes [50,51], potentially reducing the total number of hybrid gametes produced rather than their viability. Similarly, strong cytonuclear incompatibilities, which reduce hybrid viability and can prevent later generation hybrids from even entering meiosis, have been observed in crosses between species of *Saccharomyces* yeasts [52]. However, growth and sporulation rates are highly dependent on environmental conditions, and thus the contribution of these factors to reproductive isolation between yeast species is difficult to assess because they cannot be directly observed in nature.

Chromosomal mechanisms of speciation (including both rearrangements and sequence divergence) are often dismissed as unimportant because they generate underdominance [53]. Mutations that result in nondisjunction in heterozygotes (or hybrids) will be initially deleterious since rare mutants will predominantly mate with wild types. Consequently, these mutants should not rise to the frequency necessary to establish a new breeding population in which correct segregation occurs. We agree that underdominance reduces the likelihood of chromosomal mechanisms of speciation acting in obligately sexual organisms, which includes virtually all animals. However, underdominance is not necessarily a problem in facultatively sexual organisms, like yeast, in which a single mutant can rapidly establish a highly inbred population by clonal propagation. Although most speciation research is focused on obligate sexuality, facultative sexuality should not be ignored; many plants, most fungi, and nearly all unicellular eukaryotes are facultatively sexual, and this mode of reproduction is considered the ancestral state of all eukaryotes [54]. Clearly, many mechanisms can contribute to postzygotic reproductive isolation, and how these mechanisms interact to drive speciation in yeast remains an open question [55,56]. Nevertheless, the importance—to speciation in facultatively sexual organisms, at least—of small amounts of sequence divergence should not be overlooked.

## Supporting information

S1 Fig Nondisjunction frequency in interspecific hybrids measured using species-specific *YKL050c*-promoted fluorescent protein expression. Although the fluorescent signal was very weak compared to the *DIT1*-promoted fluorescent protein expression, we did attempt to score the nondisjunction frequency in interspecific N17 × Y55 hybrids for 5 different chromosome pairs (chromosome numbers indicated below points). Underlying data can be found in S1 Data. We obtained similar results with a mean nondisjunction frequency of 43.8% compared to 41.0% for the same 5 chromosomes using the *DIT1* promoter. As for the *DIT1* system, nondisjunction was extremely rare in the parents: Y55 = 0.072% (1/1,380 tetrads); N17 = 0.067% (1/1,484 tetrads).
(PDF)

S2 Fig Genetic divergence between parents is negatively correlated with spore viability in *S*. *cerevisiae* hybrids. The relationship between spore viability and parental genetic distance was investigated by Hou and colleagues [S1] for hybrids obtained by crossing various *S*. *cerevisiae* strains with S288C. (A) These data are reproduced here using the same colour scheme. That study reported ‘no apparent correlation […] between the estimated genetic divergence of the parental pairs and the resulting offspring viability […] indicating that general DNA sequence differences were not sufficient to explain the observed reproductive isolation’. In our reanalysis, we found a negative, albeit not statistically significant, correlation between hybrid spore viability and parental genetic divergence (r_s_ = −0.2578, *N* = 58, *P* = 0.0507). However, two groups (indicated by red and yellow points) represent hybrids formed between parents with different chromosomal arrangements. The red points are hybrids formed between S288C and strains carrying a Chr VIII to XVI reciprocal translocation with an *ECM34*-*SSUI* breakpoint [S1-S3]. The region on Chr VIII involved in this translocation is near the telomere and contains no essential genes, and consequently this rearrangement should cause only 25% of hybrid spores to be inviable. The yellow points represent hybrids between S288C and strains containing a translocation between two large chromosomal regions (YJM454: between the right arm of Chr V and the left arm of Chr XIV; CECT10266: between the left arm of Chr VII and the right arm of Chr XII) that each contains at least one essential gene [S1]. These two rearrangements are expected to cause a 50% reduction in spore viability in these hybrids. (B) Following the practice of Liti and colleagues [S4], we have corrected the observed spore viabilities for the effects of known chromosomal rearrangements in the groups represented by red and yellow points. The corrected spore viabilities are highly correlated with parental genetic divergence (r_s_ = −0.5289, *N* = 58, *P* < 0.0001). Thus, after correcting for the effects of chromosomal rearrangements, parental genetic divergence was a clear predictor of hybrid spore viability even among closely related parents (slope = −1.25% viability per 0.1% genetic divergence). Hybrids represented by blue points contain no known rearrangements and were therefore not adjusted. Omitting these blue points from the analysis has no effect on our conclusion (r_s_ = −0.3954, *N* = 52, *P* = 0.0037), nor does restricting the analysis to only the high-fertility collinear pairs represented by grey and blue points (r_s_ = −0.3406, *N* = 42, *P* = 0.0166). (C) Spore viabilities of intraspecific *S*. *cerevisiae* hybrids were independently measured by [S2] as part of the ‘100 genomes’ project; each strain was crossed with S288C to generate intraspecific hybrids. Since not all major chromosomal rearrangements have been identified for these strains, we have restricted our analysis to hybrids formed between parents identified as having collinear genomes (hybrid spore viability >75%) in [S2]. Full genomes for strains that Strope and colleagues [S2] provided hybrid fitness measures for but no genomic sequences were obtained from Genbank (YJM1281 = YPS163: JRIC00000000; YJM1290 = Σ1278b: JRIQ01000000; YJM1293 = RM11_1a: JRIP01000000; YJM145 = YJM789: AAFW02000000; YJM1077 = SK1: GCA_002057885.1). The genome sequence of strain YJM1628 was used for the isogenic strain YJM1615. Genetic distances were determined by aligning whole genomes using REALPHY [S5] and calculating % identity using Geneious 10.2.3 based on 8,813,278 aligned sites. Once again, hybrid spore viability was significantly correlated with parental genetic divergence (r_s_ = −0.2769, *N* = 79, *P* = 0.0135). The negative relationship was very similar to that in (B) with a slope = −1.33% viability per 0.1% genetic divergence (grey line). The slope was only slightly changed by the omission of the homozygous S288C parent (−1.35% viability per 0.1% genetic divergence). Underlying data can be found in S1 Data. (D) Predicted spore viability (red points) arising from crosses generated in this study based on NDJs (black points ± 95% confidence intervals; see S1 Data) observed for Chr I (Y55 × Y55, N17 × N17, Y55 × S288C, N17 × N44, N17 × YPS138, N17 × Y55, YPS138 × Y55, N44 × Y55, N17 × S288C). Spore viability was calculated by assuming that Chr I nondisjunction was representative for all 16 chromosomes, that spores inheriting disomes were viable, and that spores that fail to inherit a copy of one or more chromosomes are inviable. The probability of inheriting at least one copy of each chromosome is therefore equal to [(1 − NDJ) + (NDJ/2)]^16^. The dotted red line represents the relationship between spore viability and genetic divergence reported by Liti and colleagues [S4]. Our method underestimates spore viabilities for intraspecific hybrids because Chr I has a higher than average rate of nondisjunction in these strains (Fig 5). Underlying data can be found in S1 Data. Genetic divergences in panel D were taken directly from Liti and colleagues [S4]. Chr, chromosome; NDJ, nondisjunction frequency.
(PDF)

S3 Fig Recombination rate in interspecific hybrids is 1% that observed in parental types. We investigated recombination between two loci located approximately 100 kb apart on Chr XI: *YKL050c* and *YKR005c*. From genetic maps in *S*. *cerevisiae* strain S288C (https://wiki.yeastgenome.org/index.php/Combined_Physical_and_Genetic_Maps_of_S._cerevisiae), we estimated the map distance of these two loci at approximately 35 cM, the approximate limit at which linkage can be calculated by tetrad analysis without empirically derived correction [S6]. We marked *YKL050c* with P_YKL050c_-GFP_URA3 and *YKR005c* with P_DIT1_-RFP_LEU2 in both *S*. *cerevisiae* strain Y55 and *S*. *paradoxus* strain N17 and scored tetrads produced by parental diploids and hybrid diploids as the PD (2 red spores and 2 green spores), the NPD (2 red/green [represented here as yellow] spores and 2 nonfluorescent spores), or as the T (1 red/green spore, 1 red spore, 1 green spore, and 1 nonfluorescent spore). Tetrads not matching any of these three categories were omitted from the analysis (Y55 = 13, N17 = 7, Y55 × N17 = 20). Since nondisjunction is extremely rare in parental types, we calculated genetic distances in parents according to the standard equation [S6]: 100(T + 6NPD)/2(PD + NPD + T). In hybrids, the low number of Ts observed indicates that single crossover events are rare; the frequency of double crossover events must therefore be negligible, and all non-parental ditypes can be ascribed to nondisjunction events [S7]. We therefore estimated the genetic distance between these loci in hybrids as 100T/2(PD + T). Chr, chromosome; NPD, non-parental ditype; PD, parental ditype; T, tetratype.
(PDF)

S4 Fig Strain construction diagram. *LEU2* and *URA3* were knocked out using antibiotic resistance cassettes (in most cases either *HYGMX* or *KANMX*) in haploid *S*. *cerevisiae* strains Y55 and S288C and/or *S*. *paradoxus* strains (N17, N44, and YPS138) to allow selection for the integration of spore-autonomous fluorescent protein expression cassettes (tdTomato, or RFP, was linked to *LEU2*, while GFP was linked to *URA3*). Fluorescent constructs, excluding the promoters, were amplified from plasmids pSK691 (RFP_LEU2) and pSK726 (GFP_URA3) and placed under the control of the endogenous *YKL050c* or *DIT1* promoter by replacing the appropriate ORF, generating strains with ykl050c::RFP_LEU2, ykl050c::GFP_URA3, and dit1::RFP_LEU2, dit1::GFP_URA3 genomic regions. The integrated fluorescent constructs plus the appropriate endogenous strain-specific promoters (P_YKL050c_-RFP_LEU2, P_YKL050c_-GFP_URA3, P_DIT1_-RFP_LEU2, and P_DIT1_-GFP_URA3) were then amplified from genomic DNA and integrated at the desired site on each chromosome. GFP-marked haploids were mated to strains of the opposite mating type with RFP at the allelic position to generate parental or hybrid diploids, which were then sporulated to examine meiotic segregation. See S1 Text for details. GFP, green fluorescent protein; ORF, open reading frame; RFP, red fluorescent protein; tdTomato, tandem dimer Tomato fluorescent protein.
(PDF)

S1 Data Raw data used to produce figures. Data include numbers of tetrads scored as having correct segregation (different cells) or nondisjunction (same cells) for wild-type and anti-recombination–impaired (*pCLB2_SGS1*) parents and hybrids marked at each chromosome with the *DIT1*-promoted (‘DIT1 promoter’ sheet) or *YKL050c*-promoted (‘YKL050c promoter’ sheet) spore-autonomous fluorescent reporter. Tetrads with ‘other’ patterns of segregation were included in the total counts but not in the nondisjunction counts. The ‘100 genomes hybrids’ sheet includes hybrid spore viability data from [S2] and genetic distances from S288C calculated using REALPHY [S5].
(XLSX)

S1 Text Supporting materials and methods. Includes strain tables, primer tables, and supporting references.
(PDF)

## Abbreviations

Chr: chromosome
GFP: green fluorescent protein
PFGE: pulsed field gel electrophoresis
rDNA: ribosomal DNA
tdTomato: tandem dimer Tomato fluorescent protein
