# Supplementary figures and images for: Spore-autonomous fluorescent protein expression identifies meiotic chromosome mis-segregation as the principal cause of hybrid sterility in yeast
Source: PLoS Biol. 2018 Nov 12;16(11):e2005066. doi: 10.1371/journal.pbio.2005066 (PMC6258379; doi:10.1371/journal.pbio.2005066)

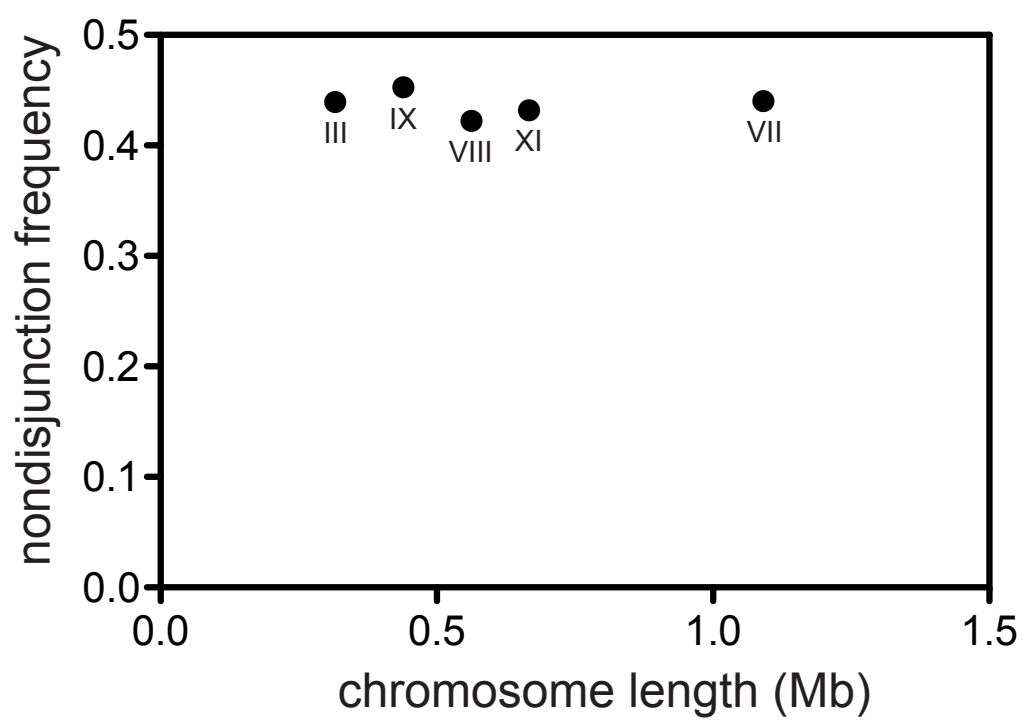

Supplement: S1 Fig — Although the fluorescent signal was very weak compared to the DIT1-promoted fluorescent protein expression, we did attempt to score the nondisjunction frequency in interspecific N17 × Y55 hybrids for 5 different chromosome pairs (chromosome numbers indicated below points). Underlying data can be found in S1 Data. We obtained similar results with a mean nondisjunction frequency of 43.8% compared to 41.0% for the same 5 chromosomes using the DIT1 promoter. As for the DIT1 system, nondisjunction was extremely rare in the parents: Y55 = 0.072% (1/1,380 tetrads); N17 = 0.067% (1/1,484 tetrads). (PDF) [file pbio.2005066.s001.pdf]

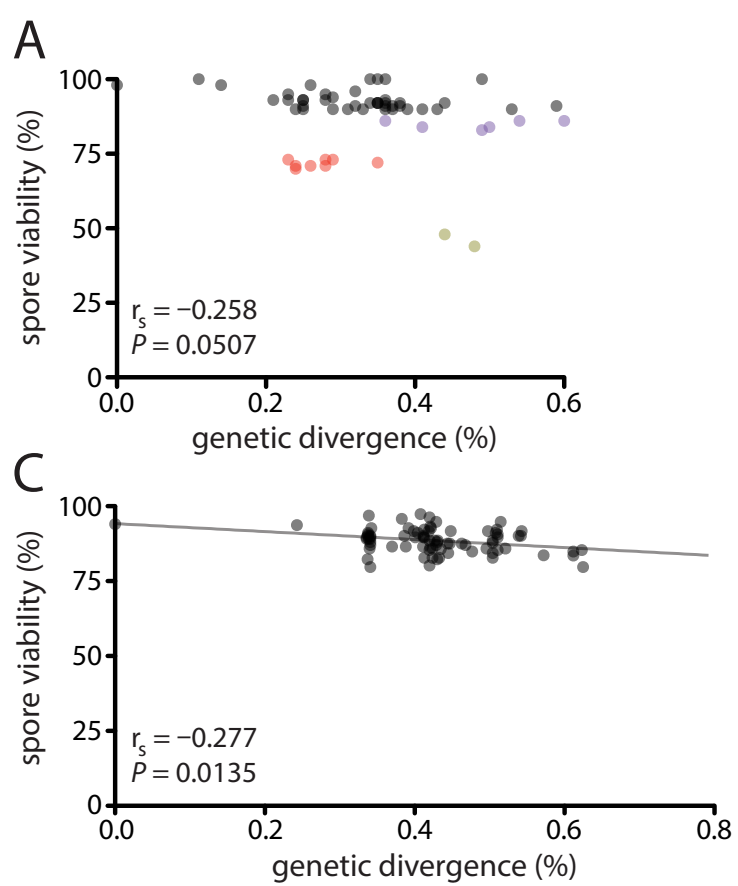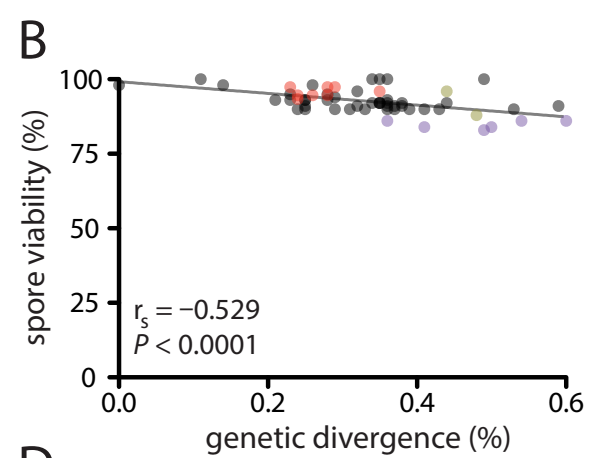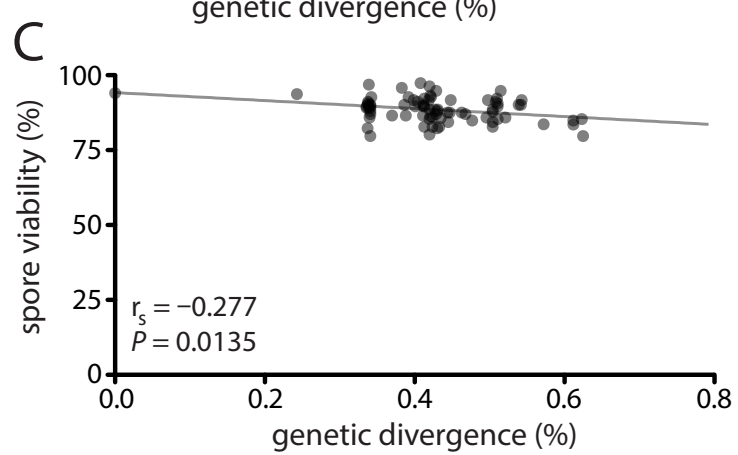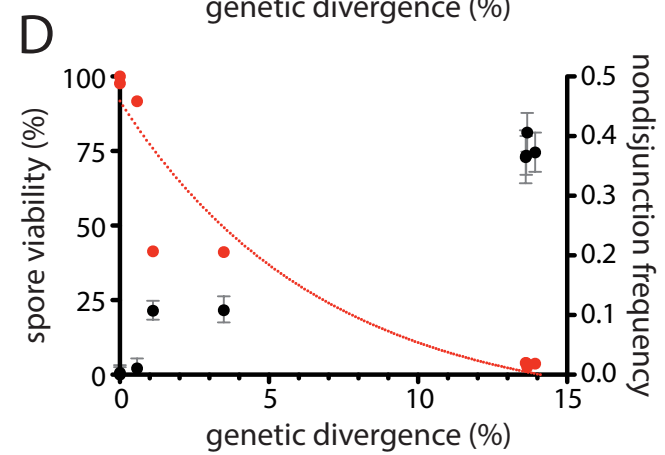

Supplement: S2 Fig — The relationship between spore viability and parental genetic distance was investigated by Hou and colleagues [S1] for hybrids obtained by crossing various S. cerevisiae strains with S288C. (A) These data are reproduced here using the same colour scheme. That study reported ‘no apparent correlation […] between the estimated genetic divergence of the parental pairs and the resulting offspring viability […] indicating that general DNA sequence differences were not sufficient to explain the observed reproductive isolation’. In our reanalysis, we found a negative, albeit not statistically significant, correlation between hybrid spore viability and parental genetic divergence (rs = −0.2578, N = 58, P = 0.0507). However, two groups (indicated by red and yellow points) represent hybrids formed between parents with different chromosomal arrangements. The red points are hybrids formed between S288C and strains carrying a Chr VIII to XVI reciprocal translocation with an ECM34-SSUI breakpoint [S1-S3]. The region on Chr VIII involved in this translocation is near the telomere and contains no essential genes, and consequently this rearrangement should cause only 25% of hybrid spores to be inviable. The yellow points represent hybrids between S288C and strains containing a translocation between two large chromosomal regions (YJM454: between the right arm of Chr V and the left arm of Chr XIV; CECT10266: between the left arm of Chr VII and the right arm of Chr XII) that each contains at least one essential gene [S1]. These two rearrangements are expected to cause a 50% reduction in spore viability in these hybrids. (B) Following the practice of Liti and colleagues [S4], we have corrected the observed spore viabilities for the effects of known chromosomal rearrangements in the groups represented by red and yellow points. The corrected spore viabilities are highly correlated with parental genetic divergence (rs = −0.5289, N = 58, P < 0.0001). Thus, after correcting for the effects of [file pbio.2005066.s002.pdf]

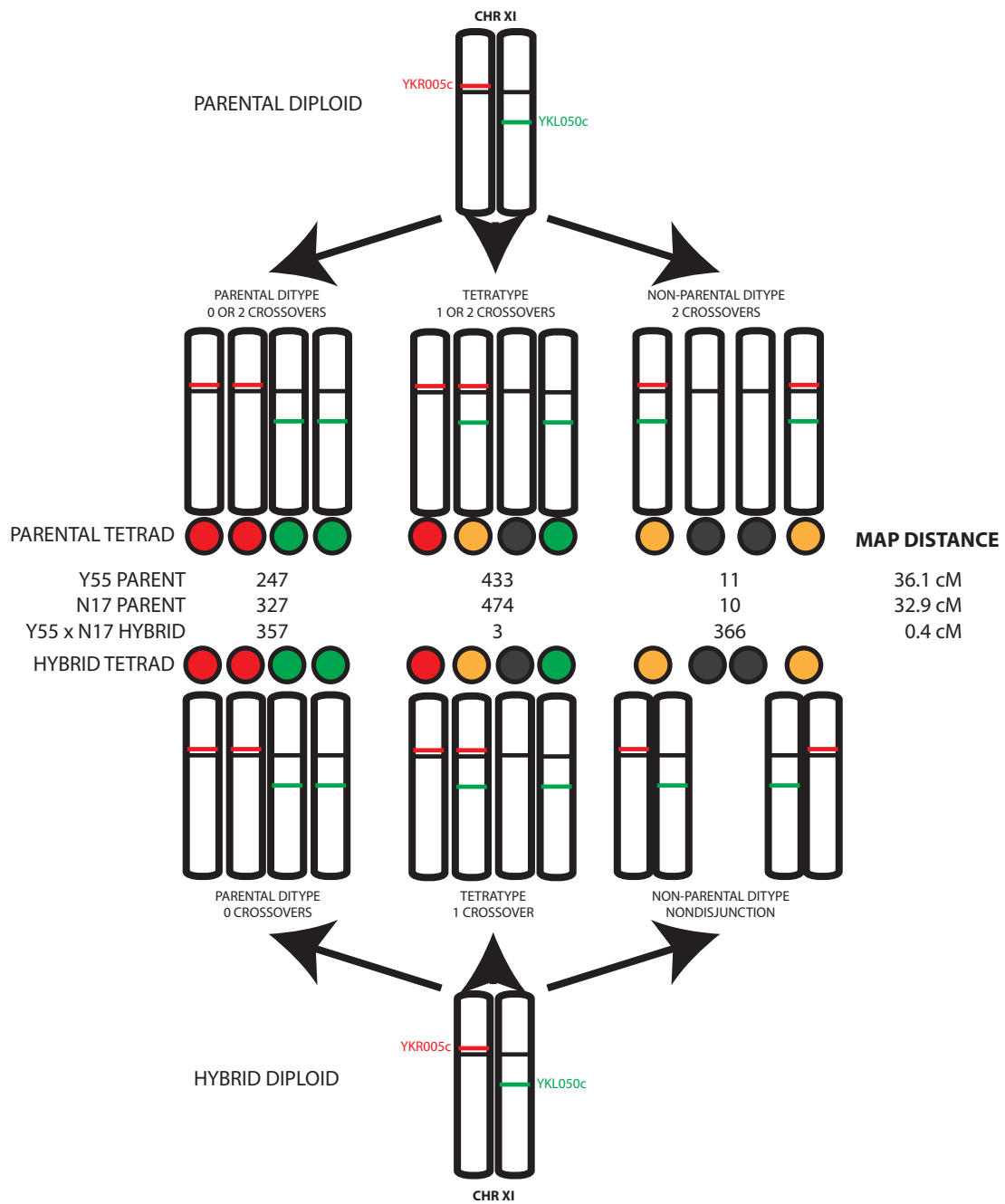

Supplement: S3 Fig — We investigated recombination between two loci located approximately 100 kb apart on Chr XI: YKL050c and YKR005c. From genetic maps in S. cerevisiae strain S288C (https://wiki.yeastgenome.org/index.php/Combined_Physical_and_Genetic_Maps_of_S._cerevisiae), we estimated the map distance of these two loci at approximately 35 cM, the approximate limit at which linkage can be calculated by tetrad analysis without empirically derived correction [S6]. We marked YKL050c with PYKL050c-GFP_URA3 and YKR005c with PDIT1-RFP_LEU2 in both S. cerevisiae strain Y55 and S. paradoxus strain N17 and scored tetrads produced by parental diploids and hybrid diploids as the PD (2 red spores and 2 green spores), the NPD (2 red/green [represented here as yellow] spores and 2 nonfluorescent spores), or as the T (1 red/green spore, 1 red spore, 1 green spore, and 1 nonfluorescent spore). Tetrads not matching any of these three categories were omitted from the analysis (Y55 = 13, N17 = 7, Y55 × N17 = 20). Since nondisjunction is extremely rare in parental types, we calculated genetic distances in parents according to the standard equation [S6]: 100(T + 6NPD)/2(PD + NPD + T). In hybrids, the low number of Ts observed indicates that single crossover events are rare; the frequency of double crossover events must therefore be negligible, and all non-parental ditypes can be ascribed to nondisjunction events [S7]. We therefore estimated the genetic distance between these loci in hybrids as 100T/2(PD + T). Chr, chromosome; NPD, non-parental ditype; PD, parental ditype; T, tetratype. (PDF) [file pbio.2005066.s003.pdf]

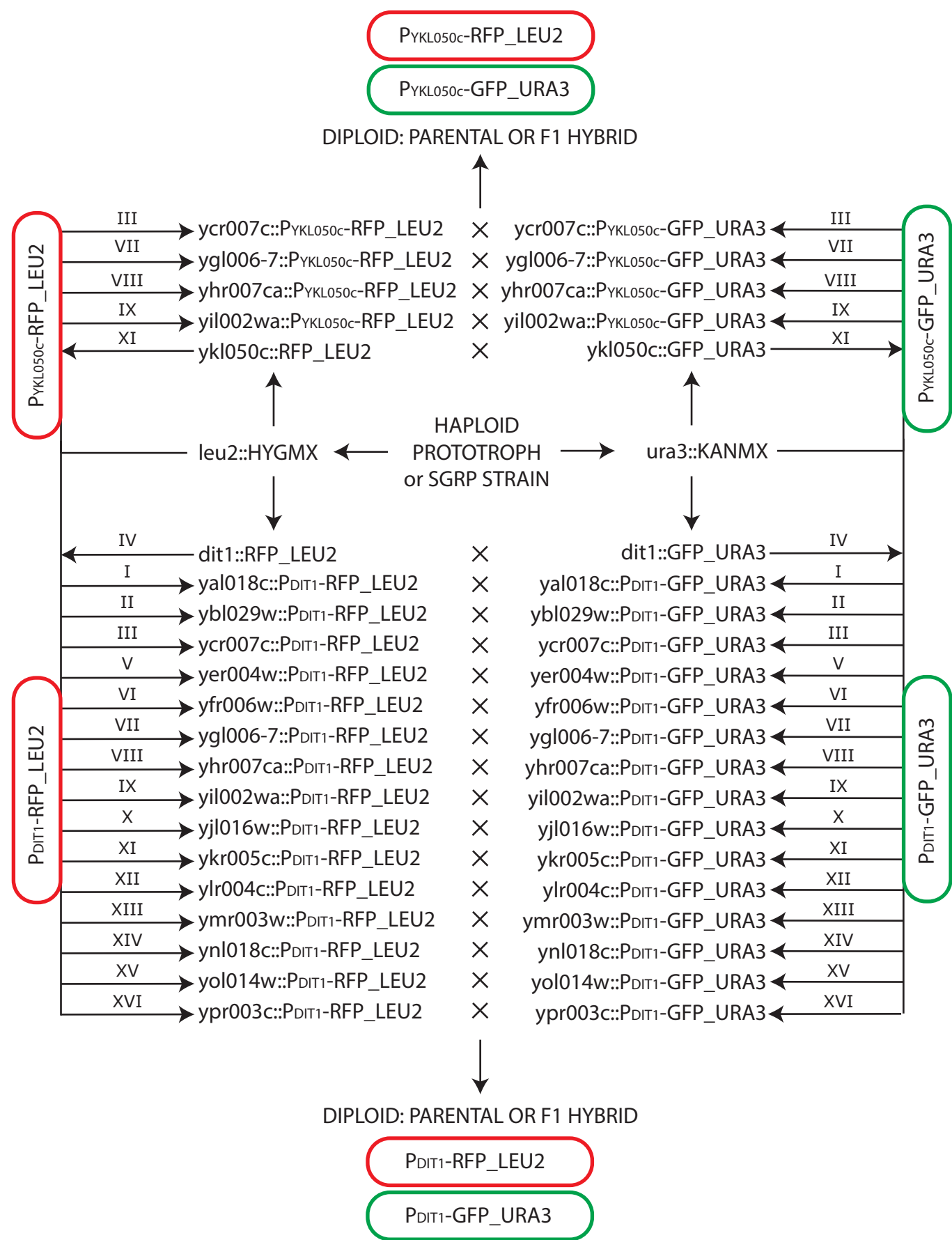

Supplement: S4 Fig — LEU2 and URA3 were knocked out using antibiotic resistance cassettes (in most cases either HYGMX or KANMX) in haploid S. cerevisiae strains Y55 and S288C and/or S. paradoxus strains (N17, N44, and YPS138) to allow selection for the integration of spore-autonomous fluorescent protein expression cassettes (tdTomato, or RFP, was linked to LEU2, while GFP was linked to URA3). Fluorescent constructs, excluding the promoters, were amplified from plasmids pSK691 (RFP_LEU2) and pSK726 (GFP_URA3) and placed under the control of the endogenous YKL050c or DIT1 promoter by replacing the appropriate ORF, generating strains with ykl050c::RFP_LEU2, ykl050c::GFP_URA3, and dit1::RFP_LEU2, dit1::GFP_URA3 genomic regions. The integrated fluorescent constructs plus the appropriate endogenous strain-specific promoters (PYKL050c-RFP_LEU2, PYKL050c-GFP_URA3, PDIT1-RFP_LEU2, and PDIT1-GFP_URA3) were then amplified from genomic DNA and integrated at the desired site on each chromosome. GFP-marked haploids were mated to strains of the opposite mating type with RFP at the allelic position to generate parental or hybrid diploids, which were then sporulated to examine meiotic segregation. See S1 Text for details. GFP, green fluorescent protein; ORF, open reading frame; RFP, red fluorescent protein; tdTomato, tandem dimer Tomato fluorescent protein. (PDF) [file pbio.2005066.s004.pdf]
