# Supplementary material for: Spore-autonomous fluorescent protein expression identifies meiotic chromosome mis-segregation as the principal cause of hybrid sterility in yeast
Source: PLoS Biol. 2018 Nov 12;16(11):e2005066. doi: 10.1371/journal.pbio.2005066 (PMC6258379; doi:10.1371/journal.pbio.2005066)
Supplement: S1 Text — Includes strain tables, primer tables, and supporting references. (PDF) [file pbio.2005066.s006.pdf]

Supporting Text

Strain construction

A diagram of the general strain construction protocol is included in S4 Fig. *S. cerevisiae* Y55 and *S. paradoxus* N17 strains were constructed starting from haploid prototrophs (Y55: MATα = YDP0302, MATa = YDP1275; N17: MATα = YDP1276, MATa = YDP0742). To allow selection for the integration of the spore-autonomous fluorescent protein expression constructs, we first knocked out the endogenous *LEU2* and *URA3* loci in one or both mating types: *LEU2* was replaced with HYGMX amplified from plasmid p167 using primers 0899/0900 (Y55: generating MATα strain YDP1307 and MATa strain YDP1303) or 0897/0898 (N17: generating MATα strain YDP1295) while *URA3* was replaced with KANMX from plasmid p161 using primers 0889/0890 (Y55: generating MATα strain YDP1277 and MATa strain YDP1285) or primers 881/882 (N17: generating MATα strain YDP1289 and MATa strain YDP1281). For *S. paradoxus* N44 and YPS138, we started with MATa haploids generated as part of the Saccharomyces Genome Resequencing Project (SGRP; [S8] that already had *URA3* replaced with KANMX and *HO* replaced with HYGMX (N44 = NCYC3687; YPS138 = NCYC3684). To allow selection of diploids formed with N17 and Y55, we swapped the ura3::KANMX marker with NATNT2 from plasmid p30346 (EUROSCARF) using primers 1057/1058 (N44: generating strain YDP1493) or 1018/1019 (YPS138: generating strain YDP1487). We subsequently replaced the ho::HYGMX markers in these strains with KANMX using primers 0255/0256 from plasmid p161 in both N44 (generating strain YDP1500) and YPS138 (generating strain YDP1470). For *S. cerevisiae* S288C, we started with MATa *ura3::*HYGMX haploid strain YDP0972, and swapped with HYGMX marker with NATMX using primers 0255/256 from plasmid p178 to generate strain YGL3.

Fluorescent proteins were placed under the expression of two different promoters. We first replaced the YKL050c ORF in Y55 and N17 with promoterless fluorescent constructs: GFP_URA3 was amplified from plasmid pSK726 and RFP_LEU2 was amplified from plasmid pSK691 (both gifts from Scott Keeney) as described in S1 Table. Genomic DNA extracted from these strains was then used as a template to transfer fluorescent constructs under the

endogenous YKL050c promoter ($P_{YKL050c}$-GFP_URA3 and $P_{YKL050c}$-RFP_LEU2) to a different chromosome (either III, VII, VIII or IX, see panel A in S1 Table). Although fluorescent protein expression under endogenous promoters was improved compared to heterologous promoters, it remained difficult to score in *S. paradoxus* and interspecific hybrids. Consequently, we switched to the *DIT1* (YDR403w) promoter, which we found could be scored with much greater reliability. The *DIT1* ORF in Y55, S288C, N17, N44, and YPS138 was replaced with a promoterless fluorescent construct as described in panel B of S1 Table. Genomic DNA from these strains was then used as a template to transfer fluorescent constructs under the *DIT1* promoter to each chromosome (except for S288C and YPS138 where we only used chromosomes I, VII, and XII). Loci chosen for integration on each chromosome were located near the centromere (but were not directly apposed to minimize influence of the fluorescent construct on centromere function), had no annotated function related to viability, meiosis, or chromosome segregation, and were not associated with reduced sporulation or germination efficiency [S9,S10]. Correct integration of fluorescent constructs at desired loci was confirmed by diagnostic PCR (primer sequences available on request).

Anti-recombination

We created meiotic null mutants of *SGS1* by replacing the endogenous promoter with the meiotically silent *CLB2* promoter, as described in [S11]. The NATNT2 cassette from plasmid p30346 (EUROSCARF) was inserted upstream of the *CLB2* promoter in Y55 strain YDP1275 (creating strain YDP1478) and in N17 strain YDP1276 (creating strain YDP1479) using primers P1013/1015 and P1012/1014, respectively. Strains YDP1478 and YDP1479 were then used as species-specific templates to replace the endogenous *SGS1* promoter with the appropriate NATNT2-linked *CLB2* promoter, using primers P1020/1021 (for Y55) and P1022/1038 (for N17), in strains with fluorescent-marked chromosomes (panel B in S1 Table). Strains of both mating types in N17 and Y55 with fluorescent markers on chromosomes II, VII, XII, and XIII (YDP1405, YDP1392, YDP1430, YDP1418, YDP1410,

YDP1397, YDP1402, YDP1389, YDP1427, YDP1415, YDP1411, YDP1398, YDP1435, YDP1436, YDP1424) were converted to *pCLB2_SGS1* (YDP1494, YDP1495, YDP1496, YDP1497, YGL13, YGL12, YDP1501, YDP1502, YDP1503, YDP1504, YDP1509, YDP1510, YGL14, YDP1511, YDP1512, respectively).

Genome alignments and % identity

Genomes for *S. cerevisiae* strains S288C and SK1 and *S. paradoxus* strains CBS432 and N44 were downloaded from https://yjx1217.github.io/Yeast_PacBio_2016/data/ and full genome alignments were performed using Mauve [S12] implemented in Geneious 10.2.3. Distances of the *S. cerevisiae* genomes from the 100-genomes project [S2] to S288C described in S2 Fig panel C and S1 Data were calculated using Geneious 10.2.3 after being aligned using REALPHY [S5].

A. *YKL050c* promoter-driven spore-autonomous fluorescence

chr	locus	Y55α		Y55a		N17α		N17a
		YDP1307	YDP1277	YDP1303	YDP1285	YDP1295	YDP1289	YDP1281
III	*YCR007c*	YDP1373<br>0948/0910<br>YDP1344			YDP1369<br>0948/0910<br>YDP1350	YDP1365<br>0947/0912<br>YDP1342		YDP1361<br>0947/0912<br>YDP1348
VII	*YGL006-7*	YDP1374<br>0935/0936<br>YDP1344			YDP1370<br>0935/0936<br>YDP1350	YDP1366<br>0933/0934<br>YDP1342		YDP1362<br>0933/0934<br>YDP1348
VIII	*YHR007cA*	YDP1375<br>0950/0908<br>YDP1344			YDP1371<br>0950/0908<br>YDP1350	YDP1367<br>0949/0906<br>YDP1342		YDP1363<br>0949/0906<br>YDP1348
IX	*YIL002wA*	YDP1376<br>0939/0940<br>YDP1344			YDP1372<br>0939/0940<br>YDP1350	YDP1368<br>0937/0938<br>YDP1342		YDP1364<br>0937/0938<br>YDP1348
XI	*YKL050c*		YDP1350<br>0920/0921<br>pSK726	YDP1344<br>0920/0921<br>pSK691	YDP1352<br>0920/0921<br>pSK726	YDP1342<br>0922/0923<br>pSK691	YDP1346<br>0922/0923<br>pSK726	YDP1348<br>0922/0923<br>pSK726

B. *DIT1* (*YDR403W*) promoter-driven spore-autonomous fluorescence

chr	locus	S288Ca	Y55α	Y55a		N17α		N17a	N44a	YPS138a
		YGL3	YDP1307	YDP1285	YDP1303	YDP1295	YDP1289	YDP1281	YDP1500	YDP1470
I	*YAL018c*	YGL8<br>1030/1031s<br>YGL5	YDP1491<br>1030/1031<br>YDP1343	YDP1490<br>1030/1031<br>YDP1351		YDP1489<br>1024/1025<br>YDP1341		YDP1488<br>1024/1025<br>YDP1347	YDP1526<br>1067/1025<br>YDP1499	YDP1513<br>1024/1025<br>YDP1498
II	*YBL029w*		YDP1389<br>0958/0959<br>YDP1343	YDP1402<br>0958/0959<br>YDP1351		YDP1415<br>0956/0957<br>YDP1341		YDP1427<br>0956/0957<br>YDP1347	YDP1527<br>1068/1069<br>YDP1499	
III	*YCR007c*		YDP1390<br>0961/0910<br>YDP1343	YDP1403<br>0961/0910<br>YDP1351		YDP1416<br>0960/0912<br>YDP1341		YDP1428<br>0960/0912<br>YDP1347	YDP1528<br>1070/1071<br>YDP1499	
IV	*YDR403w*<br>*DIT1*	YGL5<br>0916/0917<br>pSK726	YDP1349<br>0916/0917<br>pSK726	YDP1351<br>0916/0917<br>pSK726	YDP1343<br>0916/0917<br>pSK691	YDP1341<br>0918/0919<br>pSK691	YDP1345<br>0918/0919<br>pSK726	YDP1347<br>0918/0919<br>pSK726	YDP1529<br>1059/1060<br>pSK726	YDP1498<br>1036/1037<br>pSK726
V	*YER004w*		YDP1391<br>0963/0932<br>YDP1343	YDP1404<br>0963/0932<br>YDP1351		YDP1417<br>0962/0930<br>YDP1341		YDP1429<br>0962/0930<br>YDP1347	YDP1530<br>1072/0930<br>YDP1499	
VI	*YFR006w*		YDP1482<br>0966/0967<br>YDP1343	YDP1483<br>0966/0967<br>YDP1351		YDP1480<br>0964/0965<br>YDP1341		YDP1481<br>0964/0965<br>YDP1347	YDP1559<br>1097/1099<br>YDP1499	
VII	*YGL006-7*	YGL9<br>0969/0936<br>YGL5	YDP1392<br>0969/0936<br>YDP1343	YDP1405<br>0969/0936<br>YDP1351		YDP1418<br>0968/0934<br>YDP1341		YDP1430<br>0968/0934<br>YDP1347	YDP1531<br>1073/0934<br>YDP1499	YDP1521<br>1047/0934<br>YDP1498
VIII	*YHR007cA*		YDP1393<br>0971/0908<br>YDP1343	YDP1406<br>0971/0908<br>YDP1351		YDP1419<br>0970/0906<br>YDP1341		YDP1431<br>0970/0906<br>YDP1347	YDP1532<br>1074/1075<br>YDP1499	
IX	*YIL002wA*		YDP1394<br>0973/0940<br>YDP1343	YDP1407<br>0973/0940<br>YDP1351		YDP1420<br>0972/0938<br>YDP1341		YDP1432<br>0972/0938<br>YDP1347	YDP1533<br>1076/1077<br>YDP1499	
X	*YJL016w*		YDP1395<br>0976/0975<br>YDP1343	YDP1408<br>0976/0975<br>YDP1351		YDP1421<br>0974/0975<br>YDP1341		YDP1433<br>0974/0975<br>YDP1347	YDP1534<br>1078/0975<br>YDP1499	
XI	*YKR005c*		YDP1396<br>0978/0997<br>YDP1343	YDP1409<br>0978/0997<br>YDP1351		YDP1422<br>0977/0996<br>YDP1341		YDP1434<br>0977/0996<br>YDP1347	YDP1535<br>1079/1080<br>YDP1499	
XII	*YLR004c*	YGL11<br>0981/0980<br>YGL5	YDP1397<br>0981/0980<br>YDP1343	YDP1410<br>0981/0980<br>YDP1351		YDP1423<br>0979/0980<br>YDP1341		YDP1435<br>0979/0980<br>YDP1347	YDP1536<br>1081/1082<br>YDP1499	YDP1522<br>1053/0980<br>YDP1498
XIII	*YMR003w*		YDP1398<br>0984/0983<br>YDP1343	YDP1411<br>0984/0983<br>YDP1351		YDP1424<br>0982/0983<br>YDP1341		YDP1436<br>0982/0983<br>YDP1347	YDP1537<br>1083/0983<br>YDP1499	
XIV	*YNL018c*		YDP1399<br>0987/0988<br>YDP1343	YDP1412<br>0987/0988<br>YDP1351		YDP1484<br>0985/0986<br>YDP1341		YDP1485<br>0985/0986<br>YDP1347	YDP1538<br>1084/0986<br>YDP1499	
XV	*YOL014w*		YDP1400<br>0990/0998<br>YDP1343	YDP1413<br>0990/0998<br>YDP1351		YDP1425<br>0989/0998<br>YDP1341		YDP1437<br>0989/0998<br>YDP1347	YDP1539<br>1085/0998<br>YDP1499	
XVI	*YPR003c*		YDP1401<br>0993/0992<br>YDP1343	YDP1414<br>0993/0992<br>YDP1351		YDP1426<br>0991/0992<br>YDP1341		YDP1438<br>0991/0992<br>YDP1347	YDP1540<br>1086/0992<br>YDP1499	

S1 Table. Spore-autonomous fluorescent protein expression strains under (A) the *YKL050c* promoter or (B) the *DIT1* promoter. Strains are listed in red if RFP-expressing and green if

GFP-expressing and the genes into which these cassettes were integrated are listed in the locus column. *YGL006*-7 indicates the intergenic region between *YGL006W* and *YGL007W*. Primers (see S3 Table below) used for each transformation are listed below each strain number, and the source of the template DNA used in each transformation is listed below the primer numbers.

YDP0302	Y55	MATα	ho				
YDP0972	S288C	MATa	ho	ura3::HYGMX			
YGL3	S288C	MATa	ho	ura3::NATMX			
YGL5	S288C	MATa	ho	ura3::HYGMX		dit1::GFP_URA3	
YDP1307	Y55	MATα	ho		leu2::HYGMX		
YDP1275	Y55	MATa	ho				
YDP1285	Y55	MATa	ho	ura3::KANMX			
YDP1303	Y55	MATa	ho		leu2::HYGMX		
YDP1277	Y55	MATα	ho	ura3::KANMX			
YDP1349	Y55	MATα	ho	ura3::KANMX		dit1::GFP_URA3	
YDR1343	Y55	MATa	ho		leu2::HYGMX	dit1::RFP_LEU2	
YDP1285	Y55	MATa	ho				
YDP1351	Y55	MATa	ho	ura3::KANMX		dit1::GFP_URA3	
YDP1276	N17	MATα	ho				
YDP1295	N17	MATα	ho		leu2::HYGMX		
YDP1341	N17	MATα	ho		leu2::HYGMX	dit1::RFP_LEU2	
YDP1289	N17	MATα	ho	ura3::KANMX			
YDP1345	N17	MATα	ho	ura3::KANMX		dit1::GFP_URA3	
YDP0742	N17	MATa	ho				
YDP1281	N17	MATa	ho	ura3::KANMX			
YDP1347	N17	MATa	ho	ura3::KANMX		dit1::GFP_URA3	
NCYC3687	N44	MATa	ho::HYGMX	ura3::KANMX			
YDP1493	N44	MATa	ho::HYGMX	ura3::NATNT2			
YDP1499	N44	MATa	ho::HYGMX	ura3::NATNT2		dit1::GFP_URA3	
YDP1500	N44	MATa	ho::KANMX	ura3::NATNT2			
YDP1529	N44	MATa	ho::KANMX	ura3::NATNT2		dit1::GFP_URA3	
NCYC3684	YPS138	MATa	ho::HYGMX	ura3::KANMX			
YDP1487	YPS138	MATa	ho::HYGMX	ura3::NATNT2			
YDP1470	YPS138	MATa	ho::KANMX	ura3::NATNT2			
YDP1469	YPS138	MATa	ho::HYGMX	ura3::NATNT2		dit1::GFP_URA3	
YDP1498	YPS138	MATa	ho::KANMX	ura3::NATNT2		dit1::GFP_URA3	
YDP1478	Y55	MATa	ho				NATNT2_CLB2
YDP1479	N17	MATα	ho				NATNT2_CLB2

S2 Table. Starting strains used to generate spore-autonomous fluorescent protein expression strains.

primer	sequence (5'to 3')
P0255	cgtacgctgcaggtcgac
P0256	atcgatgaattcgagctcg
P0881	TGTGGCTGTGGTTTTCAGGGTCCATACTACTTTTTCTTCCcgtacgctgcaggtcgac
P0882	TTTTCGTCATTATAAAAATCATTACGACCGAGATTCCCGGatcgatgaattcgagctcg
P0889	GACCATCAAAGAAGGTTAATGTGGCTGTGGTTTCAGGGTCcgtacgctgcaggtcgac
P0890	TTTTCGTCATTATAAAAATCATTACGACCGAGATTCCCGGatcgatgaattcgagctcg
P0897	GCTATTTGGATTTTTTATATTGACTTTCATTTAACATGATcgtacgctgcaggtcgac
P0898	GCTCTACCCTATGAACATATTCCATTTTGTAATTTCGTGTatcgatgaattcgagctcg
P0899	GCTATTTGGATATTTTATATTGACTTTCGTGTACATTGATcgtacgctgcaggtcgac
P0900	CGTCTACCCTATGAACATATTCCATTTTGTAATTTCGTGTatcgatgaattcgagctcg
P0906	CCTCTCGCTTTTTGCGATACTTGCGCCTGTACACTACATTcgtttcggtgatgacggtgaaaa
P0908	TCCCTCGCTTCCTACGTTACTTGCGCCTGTGCACTACATTcgtttcggtgatgacggtgaaaa
P0910	GAAAATATCACTTTACCTGAAGACACCTTTAAATCATATAcgtttcggtgatgacggtgaaaa
P0912	GAAAATATCACTCTACCTGTAGACACCTTTAAATCATATAcgtttcggtgatgacggtgaaaa
P0916	TATCCTAATTCGGTAAAGCTTTGTCGAGACATTAACAAAAatggtgagcaagggcgagga
P0917	AAAGAACAAAAAGGTAGACCAATGTAGCGCTCTTACTTTAcgtttcggtgatgacggtgaaaa
P0918	TAATATTTTTACTCGGGAAAACTTTGAAATATTAGCAAAAatggtgagcaagggcgaggaa
P0919	AGGACAAAAAGTAAGCCGGTCTAGCGCTTCTGCTTTATCCcgtttcggtgatgacggtgaaaa
P0930	TCCCAGAAGGATTCAACGCCAAATGAACACCAACTTTTCCcgtttcggtgatgacggtgaaaa
P0932	TCCCAGAAGGATTCAACGCCAAATGAACACCAACTTTTCCcgtttcggtgatgacggtgaaaa
P0934	AAAAAGCCTCCATCGGGTTTCTAAACGGGCGTATACATGCcgtttcggtgatgacggtgaaaa
P0936	AAAAAGCCTCCATCGGGTTTTTAAACGGGCGTATACATGCcgtttcggtgatgacggtgaaaa
P0938	CGCCAAATCGTTGCTGTCCTTGTGCAGATCCTCTAACTGAcgtttcggtgatgacggtgaaaa
P0940	TGCCAAATCATTGTTGTCCCTGTGTAGATCTTCTAACTGAcgtttcggtgatgacggtgaaaa
P0956	ACATCAACGTTAATGCTTATGGTGACGAAAATTTAACGTAaggagggacaataatcgcaaagg
P0957	TGTATTCGGGGTACTTTACCCTTACTCGTTCGTGCAGTTGcgtttcggtgatgacggtgaaaa
P0958	ACATCAACGTTAATGCTTATGGTGACGAAAATTTAACGTAaggtgggacaatgattgcaaagg
P0959	TGTATTCGGGGTACTTTACCTTTACTCGTTCGTGCAGTTGcgtttcggtgatgacggtgaaaa
P0960	CCCTTTCACAAGGAATCTGAAGAATTTGTAGCACCTTTCAaggagggacaataatcgcaaagg
P0961	TTCTTTCACAAGAAATCTGAAGAATTTGTAGCATCTTTCAaggtgggacaatgattgcaaagg
P0962	CAAAGCGGATTTGGTGGCAATCTGACTGCTGCTTTGGGCAaggagggacaataatcgcaaagg
P0963	CAAAGCGGATTTGGCGGCAATCTGACTGCTGCTTTGGGCAaggtgggacaatgattgcaaagg
P0964	TTAATTGATGCAGGCGCAGAATGGAGACAATATACAAGTGaggagggacaataatcgcaaagg
P0965	GATTACCACCAACGTCGTGTACATCAAGCCCCAACATATGcgtttcggtgatgacggtgaaaa
P0966	TTAATTGATGCAGGTGCAGAATGGAGACAATATACAAGTGaggtgggacaatgattgcaaagg
P0967	GGTTACCACCAACGTCGTGTACATCAAGCCCCAACATATGcgtttcggtgatgacggtgaaaa
P0968	ATGCGCGTATATAGAGCAATTGATTGACTTAATACGCAGTaggagggacaataatcgcaaagg
P0969	ATGCGCGTATATAGAGCAATTGATTGACTTATTACGCAGTaggtgggacaatgattgcaaagg
P0970	TTACGACAGTAATACCTTCACAATTATCGGTTGGAACAATaggagggacaataatcgcaaagg
P0971	TTACGACCTTAATGGCTTTACAATTATCGGTTGGAACAATaggtgggacaatgattgcaaagg
P0972	ATGACTCGTGATACCCCTGAAGATGTCAGCACTGCAGGTGaggagggacaataatcgcaaagg
P0973	ATGACTCGTGATACACCCGAAGATGTCAGTACTGCAGGCGaggtgggacaatgattgcaaagg
P0974	TCCTCTGTACGAGTCGCAGACGTGGCCATCATAGATTCCCaggagggacaataatcgcaaagg
P0975	AATAATCCACTGAGGGCAATTTAGGTAATCCAAACATTAAcgtttcggtgatgacggtgaaaa
P0976	TCCTCTGTACAAGTCGCAGACGTGGCCATCATAGATTCCCaggtgggacaatgattgcaaagg
P0977	GACGTGATTAGGGTGGTCCTTGTTTCGGTTTCAGTCTTAGaggagggacaataatcgcaaagg
P0978	GACGTGATTAAGGTCGTCCCTGTTTCAGTATCAGTCTTGGaggtgggacaatgattgcaaagg
P0979	TAATGACACAGGAACCACTGTAAAGCAAGTACAGAGAAAAaggagggacaataatcgcaaagg
P0980	GGTCATATCACACTCATAACCACATCTATGTATATTCCAGcgtttcggtgatgacggtgaaaa
P0981	TAATGACACAGGAACCACTGTAAAGCAAGTACAGAGAAAAaggtgggacaatgattgcaaagg
P0982	AGCAGTGCAAAACAAGAGGGTTGAAATTATCAGGTCGCAAaggagggacaataatcgcaaagg
P0983	GATTCCTGAGGTTCGAGGTTCCACCAAAGACACGATAACAcgtttcggtgatgacggtgaaaa
P0984	AGCAGTGCAAATCAAGAGGGTTAAAATTATCAGGTCGCAAaggtgggacaatgattgcaaagg
P0985	TCAATGTTCTTTGTACCTCTTAGGAAGAGTGGACAGTATTaggagggacaataatcgcaaagg
P0986	AAGTCACCTGCTTTGATTTCTGCCGCACCAAGGACTCCAGcgtttcggtgatgacggtgaaaa
P0987	TCAATGTTCTTTGTACTTCTCAGGAAGAGTGGACAGTATTaggtgggacaatgattgcaaagg
P0988	AAGTGACTTGCTTTGATTTCCGCCGCACCAAGGACTCCAGcgtttcggtgatgacggtgaaaa
P0989	ACCACATTCTCAGCTGACATGCCTACTTCTTCTTGGGATAaggagggacaataatcgcaaagg
P0990	ACCACATTCTCAGCTGATATGCCAACATCTACTTGGCATGaggtgggacaatgattgcaaagg
P0991	TGTTGAGGCAAGCTTACTAAAAATTTGTCTAGCTTTAATTaggagggacaataatcgcaaagg
P0992	CCACTTGCGCTATCGTACACAACTTCAATTGCACATGTCCcgtttcggtgatgacggtgaaaa
P0993	TGTTGAGGCAAGCTTACTAAAAATTTGTCTAGCTTTAATTaggtgggacaatgattgcaaagg
P0996	TCTGCATTATTAACCTCTTTAGCCAATAATCAAAACACTAcgtttcggtgatgacggtgaaaa
P0997	TCTGCACTATTAACCTCTTTAACCAATAGTCAAAAGACTAcgtttcggtgatgacggtgaaaa
P0998	CATAATTCCTGGCATATGTTCTACCACAATTCCAATAATCcgtttcggtgatgacggtgaaaa
P1012	CCCACACTATTATCGCGCAAGAAATAATAGAAATTTATTTcgtacgctgcaggtcgac
P1013	CCTACGTTATTGTCGTGAAAGAAATGGCAGAAAATTTCTGTcgtacgctgcaggtcgac
P1014	GACAGCATAACACTTGCCCCCTGTTTTTGCAATAAGATTGcgattacaacaggtgttgtcctc
P1015	GATAACAAAACACTTGACCCCTATTTTTGTAATAAAACCGcgattacaacaggtgttgtcctc
P1018	TGTGGCTGTGGTTTTCAGGGTCCATACTACTTTTCCTTTCcgtacgctgcaggtcgac
P1019	TTTTCGTCATTATAAAAAGCATTACGACCGAGACTCCCGGcgattacaacaggtgttgtcctc
P1020	GGAAAAAAATACAGATTATTGTTGTATATATTTAAAAAATCATACACGTACACACAAGGCGGTAcgtacgctgcaggtcgac
P1021	TCGCCGTTTCCTTTAACCATTTGTGCTCCCTTCTTAAGTTATGTGACGGCTTCGTCACCATctataagatcaatgaagagagagagggg
P1022	GGAAAACTTATAAGTTCAGTGTATATATTTAAAGTCACACGCATACACGCAAGGAGGTAcgtacgctgcaggtcgac
P1024	TCACGTCTTTGTATGTTGTCGAAATTTTGTAAACGTAAAAaggagggacaataatcgcaaagg
P1025	CAAGGACGCATTCACAGGAGGTGCGTTCATGTACCCGTTCcgtttcggtgatgacggtgaaaa
P1030	TCACGTCTTTGTATGTTGTCGAAATTTTGTAAACGTAAAAaggtgggacaatgattgcaaagg
P1031	CAAAGACGCATTCACAGGAGGTGCATTCATGTACCCGTTCcgtttcggtgatgacggtgaaaa
P1031s	CAAAGATGCATTCACAGGAGGTGCATTCATGTACCCGTTCcgtttcggtgatgacggtgaaaa
P1036	CGTAAAATAAACAGTCTGTTAATATTTTTACTCGGGAAAGCTTTAAAATATTAGCAAAAatggtgagcaagggcgaggaa
P1037	ACTAACTAATATTTAAGTAAAGGACAAAAAGTAAGCCGGTCTAGCGCTGCTGCTTCATACcgtttcggtgatgacggtgaaaa
P1038	TCGCCGTTTCCTTCAACCATTTGTGCTCCCTTCTTAAGTTATGTGAGGGCTTCGTCACCATctataaaatcaatcaaaaggggatagggg
P1047	ATGCGCGTATATAGAGCAAATGATTGACTTAATACGCAATaggagggacaataatcgcaaagg
P1053	TAATGACACAGGAACCACTGTAAAGCAGGTACAGAGAAAAaggagggacaataatcgcaaagg
P1057	TGTGGCTGTGGTTTTCAGGATCCATACTACTTTTTCTTTCcgtacgctgcaggtcgac
P1058	TTTTCGTCATTATAAAAATCATTACGACCGAGATTCCCGGcgattacaacaggtgttgtcctc
P1059	TAATATTTTTACTCGGGAAAGCTTTGAAATATTAGCAAAAatggtgagcaagggcgaggaa
P1060	AAGGACAAAAAGTAAGCCGGTCTGGCGCTGCTGTTTATCCcgtttcggtgatgacggtgaaaa
P1067	TCACGTCTTTGTATGTTGTCGAAATTTTGTAAACGTAAAAaggagggacaattatcgcaaagg

P1068	ACATCAACGTTAATGCTTATGGTGACGAAAATTTAACGTAaggagggacaattatcgcaaagg
P1069	TGTATTCGGGGTACTTTACCTTTACTCGTTCGTGCAGTTGcgtttcggtgatgacggtgaaaa
P1070	CCCTTTCACAAGGAATCTGAAGAATTTGTAGCACCTTTCAaggagggacaattatcgcaaagg
P1071	GAAAATATCACTTTACCTGTAGACACCTTTAAATCATATAcgtttcggtgatgacggtgaaaa
P1072	CAAAGCGGATTTGGTGGCAATCTGACTGCTGCTTTGGGCAaggagggacaattatcgcaaagg
P1073	ATGCGCGTATATAGAGCAATTGATTGACTTAATACGCAGTaggagggacaattatcgcaaagg
P1074	TTACGACAGTAATACCTTTACAATTATCGGTTGGAACAATaggagggacaattatcgcaaagg
P1075	CCTCTCGCCTTTTGCGTTACTTGCGCCTGTACACTACATTcgtttcggtgatgacggtgaaaa
P1076	ATGACTCGTGATACCCCTGAAGATGTCAGCACTGCAGGTGaggagggacaattatcgcaaagg
P1077	CGCCAAATTGTTGCTGTCCTTGTGCAGATCCTCTAACTGAcgtttcggtgatgacggtgaaaa
P1078	TCCTCTGTACGAGTCGCAGACGTGGCCATCATAGATTCCCaggagggacaattatcgcaaagg
P1079	GACGTGATTAGGGTGGTCCTTGTTTCAGTTTCAGTCTTAGaggagggacaattatcgcaaagg
P1080	TCTGCATTATTAACCTCTTTAGCCAATGATCAAAACACTAcgtttcggtgatgacggtgaaaa
P1081	TAATGACACAGGAACCACTGTAAAGCAAGTACAGAGAAAAaggagggacaattatcgcaaagg
P1082	GGTCAAATCACACTCATAACCACATCTATGTATATTCCAGcgtttcggtgatgacggtgaaaa
P1083	AGCAGTGCAAAACAAGAGGGTTGAAATTATCAGGTCGCAAaggagggacaattatcgcaaagg
P1084	TCAATGTTCTTTGTACCTCTTAGGAAGAGTGGACAGTATTaggagggacaattatcgcaaagg
P1085	ATCACATTCTCAGCTGACATGCCTACTTCTTCTTGGGATAaggagggacaattatcgcaaagg
P1086	TGTTGAGGCAAGCTTACTAAAAATTTGTCTAGCTTTAATTaggagggacaattatcgcaaagg
P1097	TGTGGGACATTACACTACGTCAAGAATTCCGAAGATATCAaggagggacaattatcgcaaagg
P1099	CACTTGTATATTGTCTCCATTCTGCACCTGCATCAATTAAcgtttcggtgatgacggtgaaaa

S3 Table. Primers used in this study.

Supporting References

S1. Hou J, Friedrich A, De Montigny J, Schacherer J. Chromosomal rearrangements as a major mechanism in the onset of reproductive isolation in *Saccharomyces cerevisiae*. Curr Biol 2014;24:1153–9.

S2. Strope PK, Skelly DA, Kozmin SG, Mahadevan G, Stone EA, Magwene PM, et al. The 100-genomes strains, an *S. cerevisiae* resource that illuminates its natural phenotypic and genotypic variation and emergence as an opportunistic pathogen. Genome Res 2015;125:762–74.

S3. Pérez-Ortín JE, Querol A, Puig S, Barrio E. Molecular characterization of a chromosomal rearrangement involved in the edaptive evolution of yeast strains. Genome Res 2002;1533–9.

S4. Liti G, Barton DBH, Louis EJ. Sequence diversity, reproductive isolation and species concepts in saccharomyces. Genetics 2006;174:839–50.

S5. Bertels F, Silander OK, Pachkov M, Rainey PB, Van Nimwegen E. Automated reconstruction of whole-genome phylogenies from short-sequence reads. Mol Biol Evol 2014;31:1077–88.

S6. Ma C, Mortimer RK. Empirical equation that can be used to determine genetic map distances from tetrad data. Mol Cell Biol 1983; 3:1886-87.

S7. Thacker D, Lam I, Knop M, Keeney S. Exploiting spore-autonomous fluorescent protein expression to quantify meiotic chromosome behaviors in *Saccharomyces cerevisiae*. Genetics 2011;189:423–39.

S8. Cubillos FA, Louis EJ, Liti G. Generation of a large set of genetically tractable haploid and diploid *Saccharomyces* strains. FEMS Yeast Res 2009;9:1217–25.

S9. Marston AL, Tham WH, Shah H, Amon A. A genome-wide screen identifies genes required for centromeric cohesion. Science 2004;303:1367–70.

S10. Enyenihi AH, Saunders WS. Large-scale functional genomic analysis of sporulation and meiosis in *Saccharomyces cerevisiae*. Genetics 2003;163:47–54.

S11. Amin AD, Chaix ABH, Mason RP, Badge RM, Borts RH. The roles of the *Saccharomyces cerevisiae* RecQ helicase SGS1 in meiotic genome surveillance. PLoS One 2010;5:e15380.

S12. Darling ACE, Mau B, Blattner FR, Perna NT. Mauve: Multiple alignment of conserved genomic sequence with rearrangements. Genome Res 2004;14:1394-1403.
